# A maximum likelihood framework for protein design

**DOI:** 10.1186/1471-2105-7-326

**Published:** 2006-06-29

**Authors:** Claudia L Kleinman, Nicolas Rodrigue, Cécile Bonnard, Hervé Philippe, Nicolas Lartillot

**Affiliations:** 1 Canadian Institute for Advanced Research, Département de Biochimie, Université de Montréal, Montréal, Québec, Canada; 2 Laboratoire d'lnformatique, de Robotique et de Microélectronique de Montpellier, UMR 5506, CNRS-Université de Montpellier 2, 161, rue Ada, 34392 Montpellier Cedex 5, France

## Abstract

**Background:**

The aim of protein design is to predict amino-acid sequences compatible with a given target structure. Traditionally envisioned as a purely thermodynamic question, this problem can also be understood in a wider context, where additional constraints are captured by learning the sequence patterns displayed by natural proteins of known conformation. In this latter perspective, however, we still need a theoretical formalization of the question, leading to general and efficient learning methods, and allowing for the selection of fast and accurate objective functions quantifying sequence/structure compatibility.

**Results:**

We propose a formulation of the protein design problem in terms of model-based statistical inference. Our framework uses the maximum likelihood principle to optimize the unknown parameters of a statistical potential, which we call an *inverse potential *to contrast with classical potentials used for structure prediction. We propose an implementation based on Markov chain Monte Carlo, in which the likelihood is maximized by gradient descent and is numerically estimated by thermodynamic integration. The fit of the models is evaluated by cross-validation. We apply this to a simple pairwise contact potential, supplemented with a solvent-accessibility term, and show that the resulting models have a better predictive power than currently available pairwise potentials. Furthermore, the model comparison method presented here allows one to measure the relative contribution of each component of the potential, and to choose the optimal number of accessibility classes, which turns out to be much higher than classically considered.

**Conclusion:**

Altogether, this reformulation makes it possible to test a wide diversity of models, using different forms of potentials, or accounting for other factors than just the constraint of thermodynamic stability. Ultimately, such model-based statistical analyses may help to understand the forces shaping protein sequences, and driving their evolution.

## Background

Predicting the sequences compatible with a given structure defines what is traditionally called the inverse folding problem, or more often, protein design [1-3]. As suggested by the terminology, this question is usually considered in an engineering perspective: the aim is then to determine a sequence, or a set of sequences, that stably fold into a pre-specified conformation. In a thermodynamic perspective, this requirement translates into eliciting sequences that have lowest free energy under the target fold, compared to all possible alternative conformations. In principle, such a criterion would imply a search through the joint structure-sequence space, which is not feasible but for small on-lattice model proteins [4].

As an alternative to the engineering approach, a more evolutionary stance can be taken towards the inverse folding problem, in which case the aim would rather be to predict the sequences of *natural *proteins having the conformation of interest. Seen from this new point of view, the design problem raises new questions: natural proteins are the result of a complex evolutionary process, involving an intricate interplay between mutation and selection, and this probably entails many constraints directly related to the native conformation, but nevertheless not equivalent to the mere requirement of structural stability. For instance, the requirement of fast and cooperative folding has an impact on the dispersion of contact energies [5]. For this and many other potential reasons, among all sequences predicted by classical engineering-oriented protein design, probably only a subset will look like natural proteins.

The evolutionary approach to protein design is particularly relevant to phylogenetic studies, where one of the current motivations is to develop the so-called structurally constrained models of protein evolution, i.e. models explicitly dependent on the protein's conformation, either for simulation purposes [6-9], or in the context of phylogenetic inference [10,11]. In this framework, each substitution undergone by a protein during evolution has to be tested for its compatibility with the structure, in the context of the sequence that the protein displays at all other sites when the substitution occurs. Such repeated evaluation of the structure-sequence compatibility along a phylogenetic tree requires relevant and computationally very efficient scoring schemes/functions.

It is interesting to compare the different methods proposed thus far for performing protein design in light of this engineering/evolutionary distinction. A first direction of research has consisted in using all-atom semi-empirical force fields to evaluate the conformational free energy (reviewed in [12]). These empirical methods have been applied to many theoretical and experimental cases, reaching a high level of accuracy. On the other hand, they are computationally heavy, mainly because of the side-chain positioning problem, and thus cannot be easily applied to structurally constrained phylogenetic models [10,11]. Concerns may also be expressed about their over-sensitivity to the native conformation, in particular in the core of the target structures and when the flexibility of the backbone is not accounted for [13,14]. But more importantly, approaches based on physical force fields are, by definition, exclusively focussed on the conformational stability, and thereby, completely oversee other potential factors shaping the sequences of biological proteins. As such, they are well suited for engineering synthetic proteins [15], or for testing to what extent natural sequences are shaped by selection for protein stability [16], but may not be sufficient for more general evolutionary purposes.

An alternative to the semi-empirical strategy consists in relying on knowledge-based, or statistical, potentials. These scoring functions mimic physical Boltzmann distributions, but merely encode statistical patterns present in the databases. Some of these potentials were obtained under the quasi-chemical approximation, whereby frequencies of patterns, such as contacts between each pair of amino-acids, are transformed into energies using the Boltzmann law [17-20]. Alternatively, contact energies can be obtained by maximizing the potential's predictive accuracy in a threading test [21-24]. In the present context, an advantage of these knowledge-based potentials, compared to semi-empirical force-fields, is that they should in principle capture all kinds of patterns that true biological sequences have, in relation to their conformation, and not only those directly related to thermodynamic stability. Furthermore, statistical potentials need not be defined at the atomic level, but can be based on a coarse-grained description of the protein's configuration, essentially by omitting the degrees of freedom associated to side chains. This allows faster computations, by avoiding the problem of searching through the rugged landscape of side-chain conformations. In addition, coarse-grained potentials could turn out to be an advantage, in that they will not recover the native sequence too faithfully. Most protein design procedures based on statistical potentials proposed until now have relied on coarse-grained, pairwise contact pseudo-energies [4,25-32].

Yet, irrespective of the level of description adopted, currently available statistical potentials may not be ideal for protein design, since they have generally been optimized in the context of the folding problem, i.e. for maximizing the rate of correct structure prediction, given the sequence. In contrast, we would like to optimize the reciprocal prediction, namely, the sequences given the conformation. Several approaches have been proposed in this direction, consisting in maximizing the *Z*-score between the energy of the native sequence on the target conformation and its energy on a set of decoy sequences [33], or, alternatively, in applying a mean-square criterion on the values taken by the scoring function on each structure-sequence pair of the database [28]. However, these methods have thus far only been tested in cubic lattice protein models. In addition, they lack a firm theoretical basis. In particular, it would be interesting to guarantee optimal predictive power, and to have a robust methodology available to assess and compare the performance of alternative forms of statistical potentials.

Standard statistical theory provides such theoretical guarantees [34]. In the present case, the inverse folding problem can be formulated directly in terms of the probability of observing a sequence *s *given a conformation *c*, i.e. *p*(*s *| *c*, *θ*). This probability explicitly depends on the pre-specified model through a series of parameters, represented here by *θ*. These may be, for instance, the coefficients of a pairwise potential, parameters describing compositional effects, secondary structure environment, solvent accessibility, etc. Taking the product over a database of *P *independent sequence-conformation pairs, *S *= (*s*^*p*^)_*p *= 1..*P *_and *C *= (*c*^*p*^)_*p *= 1..*p*_, yields a joint probability

p(S|C,θ)=∏pp(sp|cp,θ)     (1)
 MathType@MTEF@5@5@+=feaafiart1ev1aaatCvAUfKttLearuWrP9MDH5MBPbIqV92AaeXatLxBI9gBaebbnrfifHhDYfgasaacH8akY=wiFfYdH8Gipec8Eeeu0xXdbba9frFj0=OqFfea0dXdd9vqai=hGuQ8kuc9pgc9s8qqaq=dirpe0xb9q8qiLsFr0=vr0=vr0dc8meaabaqaciaacaGaaeqabaqabeGadaaakeaacqWGWbaCdaqadaqaaiabdofatjabcYha8jabdoeadjabcYcaSGGaciab=H7aXbGaayjkaiaawMcaaiabg2da9maarafabaGaemiCaa3aaeWaaeaacqWGZbWCdaahaaWcbeqaaiabdchaWbaakiabcYha8jabdogaJnaaCaaaleqabaGaemiCaahaaOGaeiilaWIae8hUdehacaGLOaGaayzkaaaaleaacqWGWbaCaeqaniabg+GivdGccaWLjaGaaCzcamaabmaabaGaeGymaedacaGLOaGaayzkaaaaaa@4B42@

which, as a function of *θ*, can be seen as a likelihood. The parameter *θ *is then learnt by maximizing the likelihood with respect to *θ*. Once this is done, sequences can be assessed, or sampled, under the optimal parameter value θ^
 MathType@MTEF@5@5@+=feaafiart1ev1aaatCvAUfKttLearuWrP9MDH5MBPbIqV92AaeXatLxBI9gBaebbnrfifHhDYfgasaacH8akY=wiFfYdH8Gipec8Eeeu0xXdbba9frFj0=OqFfea0dXdd9vqai=hGuQ8kuc9pgc9s8qqaq=dirpe0xb9q8qiLsFr0=vr0=vr0dc8meaabaqaciaacaGaaeqabaqabeGadaaakeaadaqiaaqaaGGaciab=H7aXbGaayPadaaaaa@2F2B@, by direct numerical evaluation of their probability, or by Monte Carlo sampling methods.

Reformulated in this way, the method maximizes the predictive power of the potential, now in the structure-seeks-sequence direction. By construction, it yields the optimal parameter values that can be obtained for a given form of the potential. In addition, the fit of the model can be directly evaluated, based on the value of the likelihood obtained on a test data set, distinct from the learning set (cross-validation), giving a means of rigorous model selection. Finally, the statistical framework proposed here allows one to explicitly combine together, in a model dependent manner, all kinds of factors that we surmise may induce correlations between the structure and the sequence of proteins.

We have implemented this maximum likelihood (ML) procedure in a Markov chain Monte Carlo framework, and applied it to a simple case, using a contact potential, supplemented with a solvent accessibility term. Using cross-validation, we show that the resulting potentials yield a better fit than currently available potentials of the same form, and that combining solvent-accessibility considerations with contact energies is better than either alone. Furthermore, we find that solvent accessibility requires a more complex description than what is currently used. Ultimately, the overall method proposed in this work can be extended to a large spectrum of alternative models and statistical potentials.

## Results

### The probabilistic model

Let us consider a sequence *s *= (*s*_*i*_)_*i *= 1..*N*_, of length *N*, and of conformation *c*. In its most general form, the method introduced here can work with any model *M *specifying the conditional probability of *s *given *c*, in terms of an unnormalized non negative function *q*(*s*, *c*):

p(s|c,M)=q(s,c)∑sq(s,c).     (2)
 MathType@MTEF@5@5@+=feaafiart1ev1aaatCvAUfKttLearuWrP9MDH5MBPbIqV92AaeXatLxBI9gBaebbnrfifHhDYfgasaacH8akY=wiFfYdH8Gipec8Eeeu0xXdbba9frFj0=OqFfea0dXdd9vqai=hGuQ8kuc9pgc9s8qqaq=dirpe0xb9q8qiLsFr0=vr0=vr0dc8meaabaqaciaacaGaaeqabaqabeGadaaakeaacqWGWbaCdaqadaqaaiabdohaZjabcYha8jabdogaJjabcYcaSiabd2eanbGaayjkaiaawMcaaiabg2da9maalaaabaGaemyCae3aaeWaaeaacqWGZbWCcqGGSaalcqWGJbWyaiaawIcacaGLPaaaaeaadaaeqaqaaiabdghaXnaabmaabaGaem4CamNaeiilaWIaem4yamgacaGLOaGaayzkaaaaleaacqWGZbWCaeqaniabggHiLdaaaOGaeiOla4IaaCzcaiaaxMaadaqadaqaaiabikdaYaGaayjkaiaawMcaaaaa@4C18@

To illustrate the method, we will apply it to a simple case, using a pairwise contact potential. The argument is as follows. First, by Bayes' theorem:

p(s|c,M)=p(c|s,M)p(s|M)∑sp(c|s,M)p(s|M).     (3)
 MathType@MTEF@5@5@+=feaafiart1ev1aaatCvAUfKttLearuWrP9MDH5MBPbIqV92AaeXatLxBI9gBaebbnrfifHhDYfgasaacH8akY=wiFfYdH8Gipec8Eeeu0xXdbba9frFj0=OqFfea0dXdd9vqai=hGuQ8kuc9pgc9s8qqaq=dirpe0xb9q8qiLsFr0=vr0=vr0dc8meaabaqaciaacaGaaeqabaqabeGadaaakeaacqWGWbaCdaqadaqaaiabdohaZjabcYha8jabdogaJjabcYcaSiabd2eanbGaayjkaiaawMcaaiabg2da9maalaaabaGaemiCaa3aaeWaaeaacqWGJbWycqGG8baFcqWGZbWCcqGGSaalcqWGnbqtaiaawIcacaGLPaaacqWGWbaCdaqadaqaaiabdohaZjabcYha8jabd2eanbGaayjkaiaawMcaaaqaamaaqababaGaemiCaa3aaeWaaeaacqWGJbWycqGG8baFcqWGZbWCcqGGSaalcqWGnbqtaiaawIcacaGLPaaacqWGWbaCdaqadaqaaiabdohaZjabcYha8jabd2eanbGaayjkaiaawMcaaaWcbaGaem4CamhabeqdcqGHris5aaaakiabc6caUiaaxMaacaWLjaWaaeWaaeaacqaIZaWmaiaawIcacaGLPaaaaaa@5F64@

If, in addition, we assume a uniform prior on *s*, we can simply relate equations 3 and 2 by posing *q*(*s*, *c*) = *p*(*c *| *s*, *M*). Next, given a statistical potential *E*(*s*, *c*), the conformational probability *p*(*c *| *s*) can be expressed as a Boltzmann distribution:

p(c|s,M)=e−E(s,c)/kTZs(4)=e−(E(s,c)−F(s))/kT,(5)
 MathType@MTEF@5@5@+=feaafiart1ev1aaatCvAUfKttLearuWrP9MDH5MBPbIqV92AaeXatLxBI9gBaebbnrfifHhDYfgasaacH8akY=wiFfYdH8Gipec8Eeeu0xXdbba9frFj0=OqFfea0dXdd9vqai=hGuQ8kuc9pgc9s8qqaq=dirpe0xb9q8qiLsFr0=vr0=vr0dc8meaabaqaciaacaGaaeqabaqabeGadaaakeaafaqadeGacaaabaGaemiCaa3aaeWaaeaacqWGJbWycqGG8baFcqWGZbWCcqGGSaalcqWGnbqtaiaawIcacaGLPaaacqGH9aqpdaWcaaqaaiabdwgaLnaaCaaaleqabaGaeyOeI0Iaemyrau0aaeWaaeaacqWGZbWCcqGGSaalcqWGJbWyaiaawIcacaGLPaaacqGGVaWlcqWGRbWAcqWGubavaaaakeaacqWGAbGwdaWgaaWcbaGaem4Camhabeaaaaaakeaadaqadaqaaiabisda0aGaayjkaiaawMcaaaqaaiabg2da9iabdwgaLnaaCaaaleqabaGaeyOeI0YaaeWaaeaacqWGfbqrdaqadaqaaiabdohaZjabcYcaSiabdogaJbGaayjkaiaawMcaaiabgkHiTiabdAeagnaabmaabaGaem4CamhacaGLOaGaayzkaaaacaGLOaGaayzkaaGaei4la8Iaem4AaSMaemivaqfaaOGaeiilaWcabaWaaeWaaeaacqaI1aqnaiaawIcacaGLPaaaaaaaaa@5FA0@

where

Zs=∑ce−E(s,c)/kT     (6)
 MathType@MTEF@5@5@+=feaafiart1ev1aaatCvAUfKttLearuWrP9MDH5MBPbIqV92AaeXatLxBI9gBaebbnrfifHhDYfgasaacH8akY=wiFfYdH8Gipec8Eeeu0xXdbba9frFj0=OqFfea0dXdd9vqai=hGuQ8kuc9pgc9s8qqaq=dirpe0xb9q8qiLsFr0=vr0=vr0dc8meaabaqaciaacaGaaeqabaqabeGadaaakeaacqWGAbGwdaWgaaWcbaGaem4Camhabeaakiabg2da9maaqafabaGaemyzau2aaWbaaSqabeaacqGHsislcqWGfbqrdaqadaqaaiabdohaZjabcYcaSiabdogaJbGaayjkaiaawMcaaiabc+caViabdUgaRjabdsfaubaaaeaacqWGJbWyaeqaniabggHiLdGccaWLjaGaaCzcamaabmaabaGaeGOnaydacaGLOaGaayzkaaaaaa@43E9@

is a normalization constant, and

*F*(*s*) = - ln *Z*_*s*_.      (7)

*T *and *k *are the absolute temperature and the Boltzmann constant, respectively. Without loss of generality, it is possible to rescale the potential so that *kT *= 1, which we will do in the following.

Then, by defining the *inverse potential*:

*G*(*s*, *c*) = *E*(*s*, *c*) - *F*(*s*),      (8)

the conditional probability of sequence *s *reads as

p(s|c,θ,M)=e−G(s,c)Y,     (9)
 MathType@MTEF@5@5@+=feaafiart1ev1aaatCvAUfKttLearuWrP9MDH5MBPbIqV92AaeXatLxBI9gBaebbnrfifHhDYfgasaacH8akY=wiFfYdH8Gipec8Eeeu0xXdbba9frFj0=OqFfea0dXdd9vqai=hGuQ8kuc9pgc9s8qqaq=dirpe0xb9q8qiLsFr0=vr0=vr0dc8meaabaqaciaacaGaaeqabaqabeGadaaakeaacqWGWbaCdaqadaqaaiabdohaZjabcYha8jabdogaJjabcYcaSGGaciab=H7aXjabcYcaSiabd2eanbGaayjkaiaawMcaaiabg2da9maalaaabaGaemyzau2aaWbaaSqabeaacqGHsislcqWGhbWrdaqadaqaaiabdohaZjabcYcaSiabdogaJbGaayjkaiaawMcaaaaaaOqaaiabdMfazbaacqGGSaalcaWLjaGaaCzcamaabmaabaGaeGyoaKdacaGLOaGaayzkaaaaaa@482F@

where

Y=∑s′e−G(s′,c)     (10)
 MathType@MTEF@5@5@+=feaafiart1ev1aaatCvAUfKttLearuWrP9MDH5MBPbIqV92AaeXatLxBI9gBaebbnrfifHhDYfgasaacH8akY=wiFfYdH8Gipec8Eeeu0xXdbba9frFj0=OqFfea0dXdd9vqai=hGuQ8kuc9pgc9s8qqaq=dirpe0xb9q8qiLsFr0=vr0=vr0dc8meaabaqaciaacaGaaeqabaqabeGadaaakeaacqWGzbqwcqGH9aqpdaaeqbqaaiabdwgaLnaaCaaaleqabaGaeyOeI0Iaem4raC0aaeWaaeaacuWGZbWCgaqbaiabcYcaSiabdogaJbGaayjkaiaawMcaaaaaaeaacuWGZbWCgaqbaaqab0GaeyyeIuoakiaaxMaacaWLjaWaaeWaaeaacqaIXaqmcqaIWaamaiaawIcacaGLPaaaaaa@3FEC@

is the normalization factor. Note that, contrary to the *Z*_*s *_factor of equation 4, which was a sum over all conformations, the present factor *Y *is a sum over sequence space (all possible sequences of length *N*).

### Statistical potentials

In the present work, we used a statistical potential made of two terms:

E(s,c)=∑1≤i<j≤NΔijεsisj+∑1≤i≤Nαsivi.     (11)
 MathType@MTEF@5@5@+=feaafiart1ev1aaatCvAUfKttLearuWrP9MDH5MBPbIqV92AaeXatLxBI9gBaebbnrfifHhDYfgasaacH8akY=wiFfYdH8Gipec8Eeeu0xXdbba9frFj0=OqFfea0dXdd9vqai=hGuQ8kuc9pgc9s8qqaq=dirpe0xb9q8qiLsFr0=vr0=vr0dc8meaabaqaciaacaGaaeqabaqabeGadaaakeaacqWGfbqrdaqadaqaaiabdohaZjabcYcaSiabdogaJbGaayjkaiaawMcaaiabg2da9maaqafabaGaeuiLdq0aaSbaaSqaaiabdMgaPjabdQgaQbqabaacciGccqWF1oqzdaWgaaWcbaGaem4Cam3aaSbaaWqaaiabdMgaPbqabaWccqWGZbWCdaWgaaadbaGaemOAaOgabeaaaSqabaaabaGaeGymaeJaeyizImQaemyAaKMaeyipaWJaemOAaOMaeyizImQaemOta4eabeqdcqGHris5aOGaey4kaSYaaabuaeaacqWFXoqydaqhaaWcbaGaem4Cam3aaSbaaWqaaiabdMgaPbqabaaaleaacqWG2bGDdaWgaaadbaGaemyAaKgabeaaaaaaleaacqaIXaqmcqGHKjYOcqWGPbqAcqGHKjYOcqWGobGtaeqaniabggHiLdGccqGGUaGlcaWLjaGaaCzcamaabmaabaGaeGymaeJaeGymaedacaGLOaGaayzkaaaaaa@62BD@

The first term is a contact free energy: Δ_*ij *_= 1 if positions *i *and *j *are closer in space than a certain cut-off distance, and 0 otherwise, and *ε*_*ab *_defines the contact energy between amino acids *a *and *b*. The second term encodes a solvent-accessibility free energy: for each position, αad
 MathType@MTEF@5@5@+=feaafiart1ev1aaatCvAUfKttLearuWrP9MDH5MBPbIqV92AaeXatLxBI9gBaebbnrfifHhDYfgasaacH8akY=wiFfYdH8Gipec8Eeeu0xXdbba9frFj0=OqFfea0dXdd9vqai=hGuQ8kuc9pgc9s8qqaq=dirpe0xb9q8qiLsFr0=vr0=vr0dc8meaabaqaciaacaGaaeqabaqabeGadaaakeaaiiGacqWFXoqydaqhaaWcbaGaemyyaegabaGaemizaqgaaaaa@311B@ represents the free energy of amino acid *a *in the solvent accessibility class *d*, *a *= 1..20, and *d *= 1..*D*, where *D *is the total number of solvent accessibility classes considered.

Deriving the inverse potential requires the calculation of *F*(*s*), which is already entirely specified by the potential *E *as a sum over all conformations. However, this computation is difficult in practice. As an alternative, we can give it a simple phenomenological form, inspired from the random energy model [25,28,35]:

F(s)=−∑1≤i≤Nμsi,     (12)
 MathType@MTEF@5@5@+=feaafiart1ev1aaatCvAUfKttLearuWrP9MDH5MBPbIqV92AaeXatLxBI9gBaebbnrfifHhDYfgasaacH8akY=wiFfYdH8Gipec8Eeeu0xXdbba9frFj0=OqFfea0dXdd9vqai=hGuQ8kuc9pgc9s8qqaq=dirpe0xb9q8qiLsFr0=vr0=vr0dc8meaabaqaciaacaGaaeqabaqabeGadaaakeaacqWGgbGrdaqadaqaaiabdohaZbGaayjkaiaawMcaaiabg2da9iabgkHiTmaaqafabaacciGae8hVd02aaSbaaSqaaiabdohaZnaaBaaameaacqWGPbqAaeqaaaWcbeaakiabcYcaSaWcbaGaeGymaeJaeyizImQaemyAaKMaeyizImQaemOta4eabeqdcqGHris5aOGaaCzcaiaaxMaadaqadaqaaiabigdaXiabikdaYaGaayjkaiaawMcaaaaa@4637@

where the (*μ*_*a*_)_*a *= 1..20 _are unknown parameters, analogous to "chemical potentials" for the 20 amino acids.

Altogether, our parameter vector is made of three components: *θ *= (*α*, *ε*, *μ*), and the inverse potential reads as:



Note that the probability defined by equation 9 is invariant under the following transformation:

μ′a
 MathType@MTEF@5@5@+=feaafiart1ev1aaatCvAUfKttLearuWrP9MDH5MBPbIqV92AaeXatLxBI9gBaebbnrfifHhDYfgasaacH8akY=wiFfYdH8Gipec8Eeeu0xXdbba9frFj0=OqFfea0dXdd9vqai=hGuQ8kuc9pgc9s8qqaq=dirpe0xb9q8qiLsFr0=vr0=vr0dc8meaabaqaciaacaGaaeqabaqabeGadaaakeaaiiGacuWF8oqBgaqbamaaBaaaleaacqWGHbqyaeqaaaaa@2FEC@ = *μ*_*a *_+ *J*_1_,     (14)

ε′ab
 MathType@MTEF@5@5@+=feaafiart1ev1aaatCvAUfKttLearuWrP9MDH5MBPbIqV92AaeXatLxBI9gBaebbnrfifHhDYfgasaacH8akY=wiFfYdH8Gipec8Eeeu0xXdbba9frFj0=OqFfea0dXdd9vqai=hGuQ8kuc9pgc9s8qqaq=dirpe0xb9q8qiLsFr0=vr0=vr0dc8meaabaqaciaacaGaaeqabaqabeGadaaakeaaiiGacuWF1oqzgaqbamaaBaaaleaacqWGHbqycqWGIbGyaeqaaaaa@312A@ = *ε*_*ab *_+ *J*_2_,     (15)

α′ad
 MathType@MTEF@5@5@+=feaafiart1ev1aaatCvAUfKttLearuWrP9MDH5MBPbIqV92AaeXatLxBI9gBaebbnrfifHhDYfgasaacH8akY=wiFfYdH8Gipec8Eeeu0xXdbba9frFj0=OqFfea0dXdd9vqai=hGuQ8kuc9pgc9s8qqaq=dirpe0xb9q8qiLsFr0=vr0=vr0dc8meaabaqaciaacaGaaeqabaqabeGadaaakeaaiiGacuWFXoqygaqbamaaDaaaleaacqWGHbqyaeaacqWGKbazaaaaaa@3127@ = αad
 MathType@MTEF@5@5@+=feaafiart1ev1aaatCvAUfKttLearuWrP9MDH5MBPbIqV92AaeXatLxBI9gBaebbnrfifHhDYfgasaacH8akY=wiFfYdH8Gipec8Eeeu0xXdbba9frFj0=OqFfea0dXdd9vqai=hGuQ8kuc9pgc9s8qqaq=dirpe0xb9q8qiLsFr0=vr0=vr0dc8meaabaqaciaacaGaaeqabaqabeGadaaakeaaiiGacqWFXoqydaqhaaWcbaGaemyyaegabaGaemizaqgaaaaa@311B@ + *J*_3_,     (16)

where *J*_1_, *J*_2 _and *J*_3 _are arbitrary real constants. Therefore, to ensure identifiability of our probabilistic model, we enforce the following constraints:

∑aμa=0,     (17)
 MathType@MTEF@5@5@+=feaafiart1ev1aaatCvAUfKttLearuWrP9MDH5MBPbIqV92AaeXatLxBI9gBaebbnrfifHhDYfgasaacH8akY=wiFfYdH8Gipec8Eeeu0xXdbba9frFj0=OqFfea0dXdd9vqai=hGuQ8kuc9pgc9s8qqaq=dirpe0xb9q8qiLsFr0=vr0=vr0dc8meaabaqaciaacaGaaeqabaqabeGadaaakeaadaaeqbqaaGGaciab=X7aTnaaBaaaleaacqWGHbqyaeqaaaqaaiabdggaHbqab0GaeyyeIuoakiabg2da9iabicdaWiabcYcaSiaaxMaacaWLjaWaaeWaaeaacqaIXaqmcqaI3aWnaiaawIcacaGLPaaaaaa@3ADA@

∑abεab=0,     (18)
 MathType@MTEF@5@5@+=feaafiart1ev1aaatCvAUfKttLearuWrP9MDH5MBPbIqV92AaeXatLxBI9gBaebbnrfifHhDYfgasaacH8akY=wiFfYdH8Gipec8Eeeu0xXdbba9frFj0=OqFfea0dXdd9vqai=hGuQ8kuc9pgc9s8qqaq=dirpe0xb9q8qiLsFr0=vr0=vr0dc8meaabaqaciaacaGaaeqabaqabeGadaaakeaadaaeqbqaaGGaciab=v7aLnaaBaaaleaacqWGHbqycqWGIbGyaeqaaaqaaiabdggaHjabdkgaIbqab0GaeyyeIuoakiabg2da9iabicdaWiabcYcaSiaaxMaacaWLjaWaaeWaaeaacqaIXaqmcqaI4aaoaiaawIcacaGLPaaaaaa@3D67@

∑aαad=0, d=1..D.     (19)
 MathType@MTEF@5@5@+=feaafiart1ev1aaatCvAUfKttLearuWrP9MDH5MBPbIqV92AaeXatLxBI9gBaebbnrfifHhDYfgasaacH8akY=wiFfYdH8Gipec8Eeeu0xXdbba9frFj0=OqFfea0dXdd9vqai=hGuQ8kuc9pgc9s8qqaq=dirpe0xb9q8qiLsFr0=vr0=vr0dc8meaabaqaciaacaGaaeqabaqabeGadaaakeaadaaeqbqaaGGaciab=f7aHnaaDaaaleaacqWGHbqyaeaacqWGKbazaaaabaGaemyyaegabeqdcqGHris5aOGaeyypa0JaeGimaaJaeiilaWIaeeiiaaIaemizaqMaeyypa0JaeGymaeJaeiOla4IaeiOla4IaemiraqKaeiOla4IaaCzcaiaaxMaadaqadaqaaiabigdaXiabiMda5aGaayjkaiaawMcaaaaa@43E4@

A series of alternative inverse potentials can be obtained by suppressing the first or the second of the components of equation 13. In the present work, we tested the following combinations:

• *μ*,

• *α *+ *μ*,

• *ε *+ *μ*,

• *ε *+ *α *+ *μ*.

We also explored various numbers of accessibility classes, with *D *ranging from 2 to 20. Alternatively, the *ε *component can be fixed to values of a contact potential obtained by other authors (MJ) [17]. In this case, we must add a multiplicative scaling factor *λ *in front of the contact component to account for the fact that these potentials are normalized differently:



The scaling factor is optimized by ML, along with *μ*.

### Optimizing the potentials by gradient descent

If we now consider a database, made of *P *protein sequences *S *= (*s*^*p*^)_*p *= 1..*P*_, of respective lengths *N*_*p *_and their corresponding three dimensional structures *C *= (*c*^*p*^)_*p *= 1..*P*_, the probability of observing the whole database, which we define as the *likelihood L*(*θ*), is the product of the probabilities of observing each protein independently:

L(θ)=p(S|C,θ)(21)=∏pp(sp|cp,θ)(22)=e−G(S,C)Y(23)
 MathType@MTEF@5@5@+=feaafiart1ev1aaatCvAUfKttLearuWrP9MDH5MBPbIqV92AaeXatLxBI9gBaebbnrfifHhDYfgasaacH8akY=wiFfYdH8Gipec8Eeeu0xXdbba9frFj0=OqFfea0dXdd9vqai=hGuQ8kuc9pgc9s8qqaq=dirpe0xb9q8qiLsFr0=vr0=vr0dc8meaabaqaciaacaGaaeqabaqabeGadaaakeaafaqadeWacaaabaGaemitaW0aaeWaaeaaiiGacqWF4oqCaiaawIcacaGLPaaacqGH9aqpcqWGWbaCdaqadaqaaiabdofatjabcYha8jabdoeadjabcYcaSiab=H7aXbGaayjkaiaawMcaaaqaamaabmaabaGaeGOmaiJaeGymaedacaGLOaGaayzkaaaabaGaeyypa0ZaaebuaeaacqWGWbaCdaqadaqaaiabdohaZnaaCaaaleqabaGaemiCaahaaOGaeiiFaWNaem4yam2aaWbaaSqabeaacqWGWbaCaaGccqGGSaalcqWF4oqCaiaawIcacaGLPaaaaSqaaiabdchaWbqab0Gaey4dIunaaOqaamaabmaabaGaeGOmaiJaeGOmaidacaGLOaGaayzkaaaabaGaeyypa0ZaaSaaaeaacqWGLbqzdaahaaWcbeqaaiabgkHiTiabdEeahnaabmaabaGaem4uamLaeiilaWIaem4qameacaGLOaGaayzkaaaaaaGcbaGaemywaKfaaaqaamaabmaabaGaeGOmaiJaeG4mamdacaGLOaGaayzkaaaaaaaa@61C8@

where

G(S,C)=∑pG(sp,cp)     (24)
 MathType@MTEF@5@5@+=feaafiart1ev1aaatCvAUfKttLearuWrP9MDH5MBPbIqV92AaeXatLxBI9gBaebbnrfifHhDYfgasaacH8akY=wiFfYdH8Gipec8Eeeu0xXdbba9frFj0=OqFfea0dXdd9vqai=hGuQ8kuc9pgc9s8qqaq=dirpe0xb9q8qiLsFr0=vr0=vr0dc8meaabaqaciaacaGaaeqabaqabeGadaaakeaacqWGhbWrdaqadaqaaiabdofatjabcYcaSiabdoeadbGaayjkaiaawMcaaiabg2da9maaqafabaGaem4raC0aaeWaaeaacqWGZbWCdaahaaWcbeqaaiabdchaWbaakiabcYcaSiabdogaJnaaCaaaleqabaGaemiCaahaaaGccaGLOaGaayzkaaaaleaacqWGWbaCaeqaniabggHiLdGccaWLjaGaaCzcamaabmaabaGaeGOmaiJaeGinaqdacaGLOaGaayzkaaaaaa@4539@

is the inverse potential summed over the database, and

Y=∑S′e−G(S′,C)     (25)
 MathType@MTEF@5@5@+=feaafiart1ev1aaatCvAUfKttLearuWrP9MDH5MBPbIqV92AaeXatLxBI9gBaebbnrfifHhDYfgasaacH8akY=wiFfYdH8Gipec8Eeeu0xXdbba9frFj0=OqFfea0dXdd9vqai=hGuQ8kuc9pgc9s8qqaq=dirpe0xb9q8qiLsFr0=vr0=vr0dc8meaabaqaciaacaGaaeqabaqabeGadaaakeaacqWGzbqwcqGH9aqpdaaeqbqaaiabdwgaLnaaCaaaleqabaGaeyOeI0Iaem4raC0aaeWaaeaacuWGtbWugaqbaiabcYcaSiabdoeadbGaayjkaiaawMcaaaaaaeaacuWGtbWugaqbaaqab0GaeyyeIuoakiaaxMaacaWLjaWaaeWaaeaacqaIYaGmcqaI1aqnaiaawIcacaGLPaaaaaa@3F38@

is the corresponding normalization constant. Since it is more convenient to work on minus the logarithm of the probability, we define the score *ω*:

*ω*(*θ*) = - ln *L*(*θ*)      (26)

= *G*(*S*, *C*) + ln *Y*.      (27)

We wish to maximize the likelihood, or equivalently, minimize *ω*, with respect to *θ*. We do this by gradient descent, based on a numerical evaluation of the derivative of *ω *(see methods). The overall method is akin to an Expectation Maximization algorithm [36]. In fact, it can be seen as a differential version of Dempster's method, and therefore, we call it *differential EM*.

The derivative of *ω *reads as:

∂ω∂θ=∂G(S,C)∂θ+∂ln⁡Y∂θ.     (28)
 MathType@MTEF@5@5@+=feaafiart1ev1aaatCvAUfKttLearuWrP9MDH5MBPbIqV92AaeXatLxBI9gBaebbnrfifHhDYfgasaacH8akY=wiFfYdH8Gipec8Eeeu0xXdbba9frFj0=OqFfea0dXdd9vqai=hGuQ8kuc9pgc9s8qqaq=dirpe0xb9q8qiLsFr0=vr0=vr0dc8meaabaqaciaacaGaaeqabaqabeGadaaakeaadaWcaaqaaGGaciab=jGi2kab=L8a3bqaaiab=jGi2kab=H7aXbaacqGH9aqpdaWcaaqaaiab=jGi2kabdEeahnaabmaabaGaem4uamLaeiilaWIaem4qameacaGLOaGaayzkaaaabaGae8NaIyRae8hUdehaaiabgUcaRmaalaaabaGae8NaIyRagiiBaWMaeiOBa4MaemywaKfabaGae8NaIyRae8hUdehaaiabc6caUiaaxMaacaWLjaWaaeWaaeaaieaacqGFYaGmcqGFZaWmaiaawIcacaGLPaaaaaa@4D30@

Applying the partition function formalism to equation 25, we can express the second term as an expectation over *p*(*S*' | *C*, *θ*):

∂ln⁡Y∂θ=1Y∂Y∂θ(29)=−1Y∑S′∂G(S′,C)∂θe−G(S′,C)(30)=−∑S′∂G(S′,C)∂θp(S′|C,θ)(31)=−〈∂G∂θ〉(32)
 MathType@MTEF@5@5@+=feaafiart1ev1aaatCvAUfKttLearuWrP9MDH5MBPbIqV92AaeXatLxBI9gBaebbnrfifHhDYfgasaacH8akY=wiFfYdH8Gipec8Eeeu0xXdbba9frFj0=OqFfea0dXdd9vqai=hGuQ8kuc9pgc9s8qqaq=dirpe0xb9q8qiLsFr0=vr0=vr0dc8meaabaqaciaacaGaaeqabaqabeGadaaakeaafaqadeabcaaaaeaadaWcaaqaaGGaciab=jGi2kGbcYgaSjabc6gaUjabdMfazbqaaiab=jGi2kab=H7aXbaacqGH9aqpdaWcaaqaaiabigdaXaqaaiabdMfazbaadaWcaaqaaiab=jGi2kabdMfazbqaaiab=jGi2kab=H7aXbaaaeaadaqadaqaaiabikdaYiabiMda5aGaayjkaiaawMcaaaqaaiabg2da9iabgkHiTmaalaaabaGaeGymaedabaGaemywaKfaamaaqafabaWaaSaaaeaacqWFciITcqWGhbWrdaqadaqaaiqbdofatzaafaGaeiilaWIaem4qameacaGLOaGaayzkaaaabaGae8NaIyRae8hUdehaaaWcbaGafm4uamLbauaaaeqaniabggHiLdGccqWGLbqzdaahaaWcbeqaaiabgkHiTiabdEeahnaabmaabaGafm4uamLbauaacqGGSaalcqWGdbWqaiaawIcacaGLPaaaaaaakeaadaqadaqaaiabiodaZiabicdaWaGaayjkaiaawMcaaaqaaiabg2da9iabgkHiTmaaqafabaWaaSaaaeaacqWFciITcqWGhbWrdaqadaqaaiqbdofatzaafaGaeiilaWIaem4qameacaGLOaGaayzkaaaabaGae8NaIyRae8hUdehaaaWcbaGafm4uamLbauaaaeqaniabggHiLdGccqWGWbaCdaqadaqaaiqbdofatzaafaGaeiiFaWNaem4qamKaeiilaWIae8hUdehacaGLOaGaayzkaaaabaWaaeWaaeaacqaIZaWmcqaIXaqmaiaawIcacaGLPaaaaeaacqGH9aqpcqGHsislcqGHPms4daWcaaqaaiab=jGi2kabdEeahbqaaiab=jGi2kab=H7aXbaacqGHQms8aeaadaqadaqaaiabiodaZiabikdaYaGaayjkaiaawMcaaaaaaaa@8A2A@

which leads us to the following expression for the derivative of *ω*:

∂ω∂θ=∂G(S,C)∂θ−〈∂G∂θ〉.     (33)
 MathType@MTEF@5@5@+=feaafiart1ev1aaatCvAUfKttLearuWrP9MDH5MBPbIqV92AaeXatLxBI9gBaebbnrfifHhDYfgasaacH8akY=wiFfYdH8Gipec8Eeeu0xXdbba9frFj0=OqFfea0dXdd9vqai=hGuQ8kuc9pgc9s8qqaq=dirpe0xb9q8qiLsFr0=vr0=vr0dc8meaabaqaciaacaGaaeqabaqabeGadaaakeaadaWcaaqaaGGaciab=jGi2kab=L8a3bqaaiab=jGi2kab=H7aXbaacqGH9aqpdaWcaaqaaiab=jGi2kabdEeahnaabmaabaGaem4uamLaeiilaWIaem4qameacaGLOaGaayzkaaaabaGae8NaIyRae8hUdehaaiabgkHiTiabgMYiHpaalaaabaGae8NaIyRaem4raCeabaGae8NaIyRae8hUdehaaiabgQYiXlabc6caUiaaxMaacaWLjaWaaeWaaeaacqaIZaWmcqaIZaWmaiaawIcacaGLPaaaaaa@4DE1@

The computation of the first term in this equation is straightforward, while the second term must be estimated numerically. In order to do so, we obtain a sample  drawn from *p*(*S *| *C*, *θ*) by a Gibbs sampling algorithm similar to that of Robinson et al. [10] (see methods).

Applying formula 33 on the inverse potential 13 yields the following expressions for the derivatives:

∂ω∂εab=−[nab−〈nab〉],     (34)
 MathType@MTEF@5@5@+=feaafiart1ev1aaatCvAUfKttLearuWrP9MDH5MBPbIqV92AaeXatLxBI9gBaebbnrfifHhDYfgasaacH8akY=wiFfYdH8Gipec8Eeeu0xXdbba9frFj0=OqFfea0dXdd9vqai=hGuQ8kuc9pgc9s8qqaq=dirpe0xb9q8qiLsFr0=vr0=vr0dc8meaabaqaciaacaGaaeqabaqabeGadaaakeaadaWcaaqaaGGaciab=jGi2kab=L8a3bqaaiab=jGi2kab=v7aLnaaBaaaleaacqWGHbqycqWGIbGyaeqaaaaakiabg2da9iabgkHiTmaadmaabaGaemOBa42aaSbaaSqaaiabdggaHjabdkgaIbqabaGccqGHsisldaaadaqaaiabd6gaUnaaBaaaleaacqWGHbqycqWGIbGyaeqaaaGccaGLPmIaayPkJaaacaGLBbGaayzxaaGaeiilaWIaaCzcaiaaxMaadaqadaqaaiabiodaZiabisda0aGaayjkaiaawMcaaaaa@4A5D@

where *n*_*ab *_is the number of contacts between amino acids *a *and *b *observed in the database, and 〈*n*_*ab*_〉 is its expectation over the probability distribution *p*(*S*' | *C*, *θ*). Formula 34 thus leads to an intuitive characterization of the maximum likelihood estimate ε^
 MathType@MTEF@5@5@+=feaafiart1ev1aaatCvAUfKttLearuWrP9MDH5MBPbIqV92AaeXatLxBI9gBaebbnrfifHhDYfgasaacH8akY=wiFfYdH8Gipec8Eeeu0xXdbba9frFj0=OqFfea0dXdd9vqai=hGuQ8kuc9pgc9s8qqaq=dirpe0xb9q8qiLsFr0=vr0=vr0dc8meaabaqaciaacaGaaeqabaqabeGadaaakeaadaqiaaqaaGGaciab=v7aLbGaayPadaaaaa@2F1C@: it is the value of *ε *such that the average number of each type of contact predicted by the potential matches the number observed in the database. Following a similar derivation:

∂ω∂μa=−[ma−〈ma〉],     (35)
 MathType@MTEF@5@5@+=feaafiart1ev1aaatCvAUfKttLearuWrP9MDH5MBPbIqV92AaeXatLxBI9gBaebbnrfifHhDYfgasaacH8akY=wiFfYdH8Gipec8Eeeu0xXdbba9frFj0=OqFfea0dXdd9vqai=hGuQ8kuc9pgc9s8qqaq=dirpe0xb9q8qiLsFr0=vr0=vr0dc8meaabaqaciaacaGaaeqabaqabeGadaaakeaadaWcaaqaaGGaciab=jGi2kab=L8a3bqaaiab=jGi2kab=X7aTnaaBaaaleaacqWGHbqyaeqaaaaakiabg2da9iabgkHiTmaadmaabaGaemyBa02aaSbaaSqaaiabdggaHbqabaGccqGHsisldaaadaqaaiabd2gaTnaaBaaaleaacqWGHbqyaeqaaaGccaGLPmIaayPkJaaacaGLBbGaayzxaaGaeiilaWIaaCzcaiaaxMaadaqadaqaaiabiodaZiabiwda1aGaayjkaiaawMcaaaaa@4683@

where *m*_*a *_is the total number of amino acids of type *a*, and

∂ω∂αad=−[lad−〈lad〉],     (36)
 MathType@MTEF@5@5@+=feaafiart1ev1aaatCvAUfKttLearuWrP9MDH5MBPbIqV92AaeXatLxBI9gBaebbnrfifHhDYfgasaacH8akY=wiFfYdH8Gipec8Eeeu0xXdbba9frFj0=OqFfea0dXdd9vqai=hGuQ8kuc9pgc9s8qqaq=dirpe0xb9q8qiLsFr0=vr0=vr0dc8meaabaqaciaacaGaaeqabaqabeGadaaakeaadaWcaaqaaGGaciab=jGi2kab=L8a3bqaaiab=jGi2kab=f7aHnaaDaaaleaacqWGHbqyaeaacqWGKbazaaaaaOGaeyypa0JaeyOeI0YaamWaaeaacqWGSbaBdaqhaaWcbaGaemyyaegabaGaemizaqgaaOGaeyOeI0YaaaWaaeaacqWGSbaBdaqhaaWcbaGaemyyaegabaGaemizaqgaaaGccaGLPmIaayPkJaaacaGLBbGaayzxaaGaeiilaWIaaCzcaiaaxMaadaqadaqaaiabiodaZiabiAda2aGaayjkaiaawMcaaaaa@4A60@

where lad
 MathType@MTEF@5@5@+=feaafiart1ev1aaatCvAUfKttLearuWrP9MDH5MBPbIqV92AaeXatLxBI9gBaebbnrfifHhDYfgasaacH8akY=wiFfYdH8Gipec8Eeeu0xXdbba9frFj0=OqFfea0dXdd9vqai=hGuQ8kuc9pgc9s8qqaq=dirpe0xb9q8qiLsFr0=vr0=vr0dc8meaabaqaciaacaGaaeqabaqabeGadaaakeaacqWGSbaBdaqhaaWcbaGaemyyaegabaGaemizaqgaaaaa@30D6@ is the total number of amino acids of type *a *belonging to solvent-accessibility class *d*.

**Figure 1:**
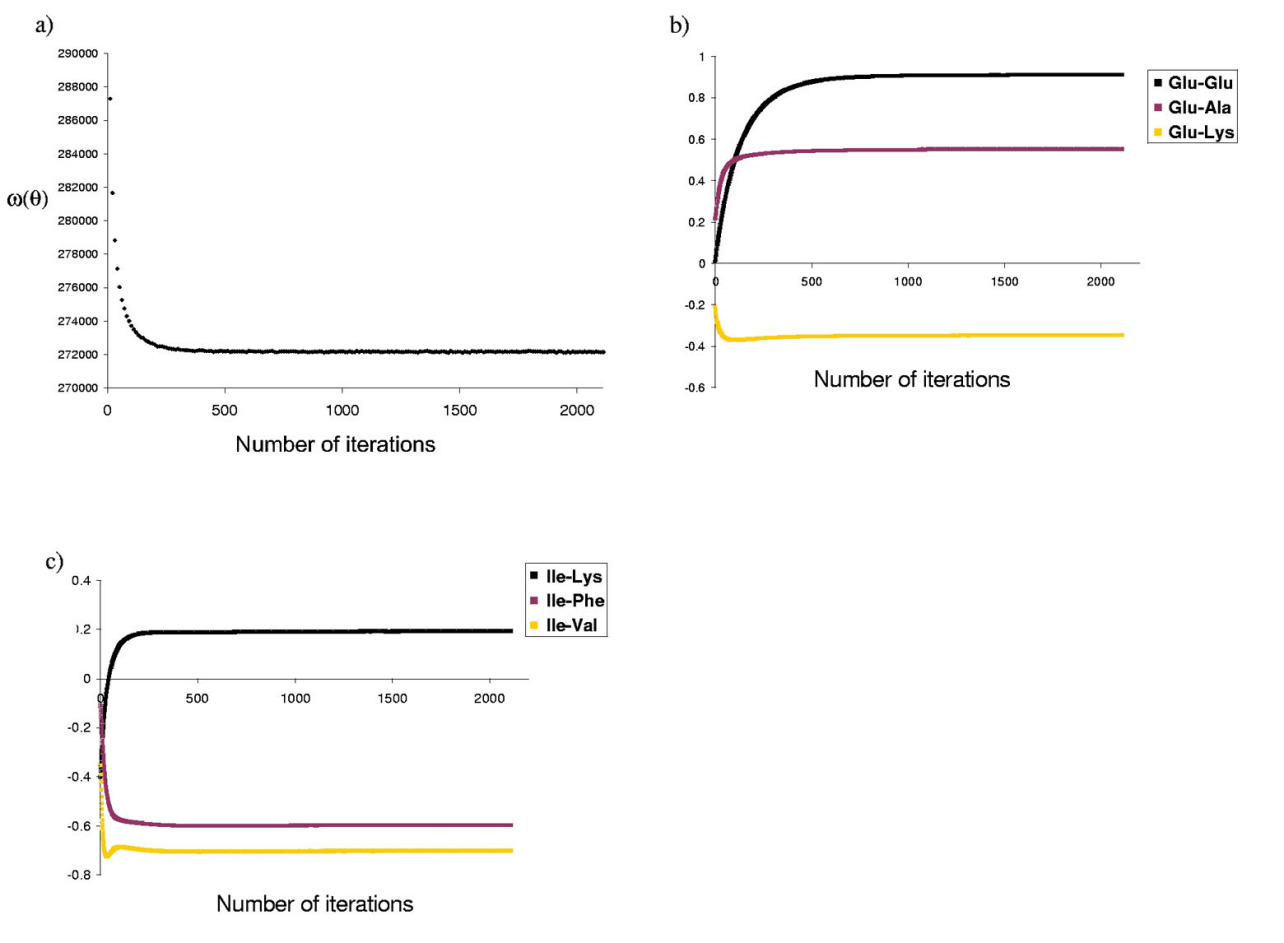
**Convergence of the optimization procedure**. Traceplots illustrating the convergence of the differential EM method in the optimization of contact potentials, on data set DS1. Are shown, as a function of the number of iterations **(a) **the score *ω*(*θ*) = In *p*(*S *| *C*, *θ*), **(b) **and **(c) **examples of pairwise contact energies obtained for some amino acid pairs.

We first performed an optimization of the pure contact potential (*ε *+ *μ*-potential) on each data set. Figure 1 shows the evolution of the scoring function *ω *and of the contact potential during the gradient descent. As can be seen from these traceplots, the differential EM algorithm converges after a few hundred cycles. The scoring function stabilizes at around 272,000 natural units of logarithm (nits), and then fluctuates by up to 25 nits around this value. These fluctuations are mainly due to the finite size of the sample of sequences on which the derivative of ln *Y *is evaluated and, to a lesser extent, to the error on the estimation of ln *Y *by thermodynamic integration. In any case, these errors are small compared to the differences between scores obtained with alternative models (see below).

The evolution of the potential for some residue pairs is shown in figure 1b and 1c. Effects in the final values due to residue polarity are easily seen: known favorable interactions such as glutamate-lysine or the hydrophobic isoleucine-valine have a lower contact energy, while known unfavorable interactions, such as glutamate-glutamate, have higher energies, indicating that the potentials obtained are biologically reasonable.

**Figure 2:**
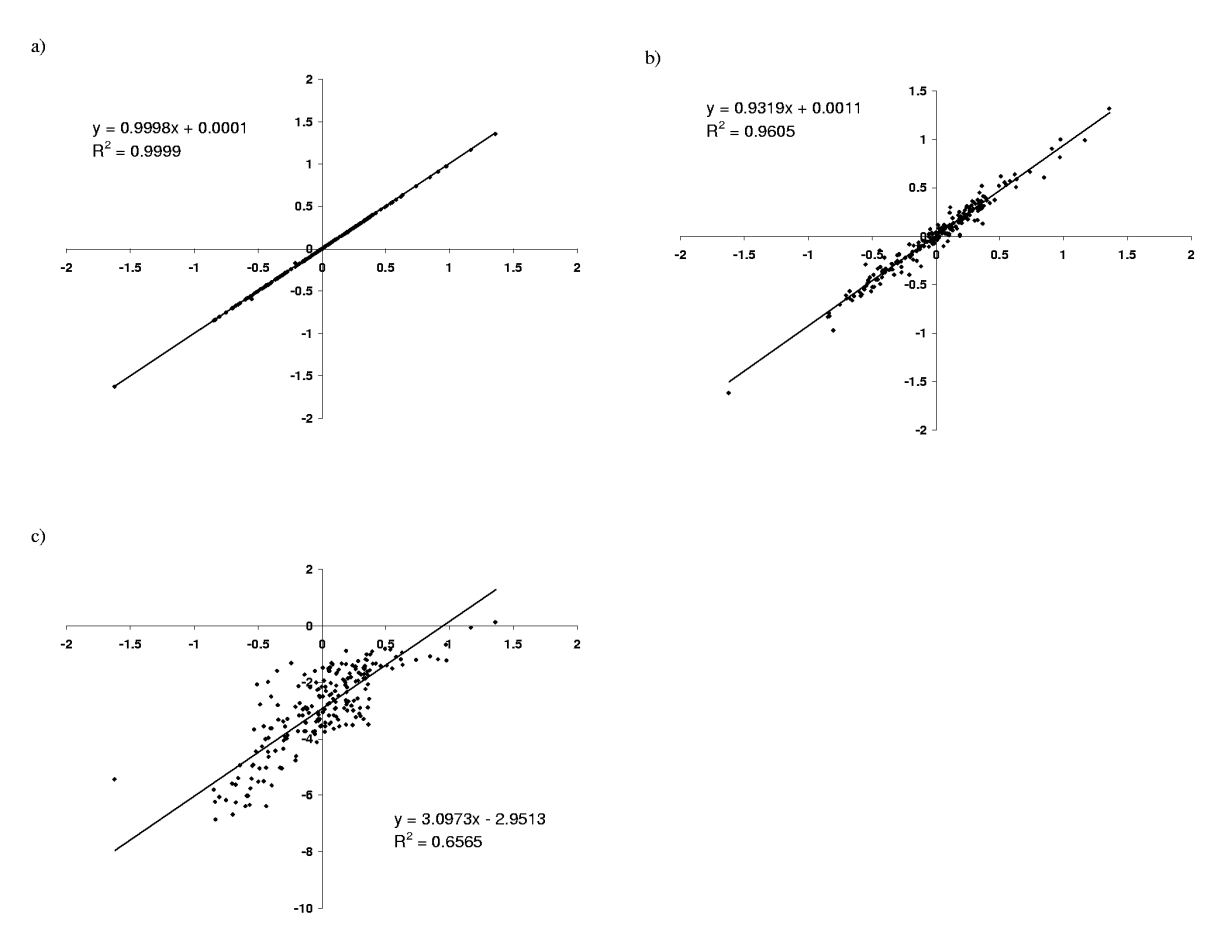
**XY-comparisons of pairwise contact potentials**. **(a) **two independent runs on the same data set DS1, **(b) **two runs, on data sets DS1 (X-axis) and DS2 (Y-axis); **(c) **Miyazawa and Jernigan's potential, compared to that obtained on DS1.

The potentials obtained in two independent runs are virtually identical (figure 2a), indicating that the gradient descent does not get trapped into local minima. We can also compare the values of the potential for two distinct data sets of equivalent size, DS1 and DS2 (figure 2b), which uncovers a greater discrepancy than for two independent runs on the same data set DS1. The correlation is high, however, suggesting that data sets are large enough for the learning procedure to reach stability. In addition, these differences are small compared to the discrepancy between the potential obtained by our method and that of Miyazawa & Jernigan (figure 2c).

### Model comparison

The same optimization procedure was applied to the potential consisting only of the solvent accessibility term (*α *+ *μ*), with an increasing number of accessibility classes, and to the combined (*ε *+ *α *+ *μ*) potential. The resulting log likelihood scores cannot directly be compared, since the models do not have the same dimensionality. We therefore applied a 2-fold cross-validation procedure (CV), consisting in learning the potential on DS2, and testing it on DS1, and vice versa.

**Figure 3:**
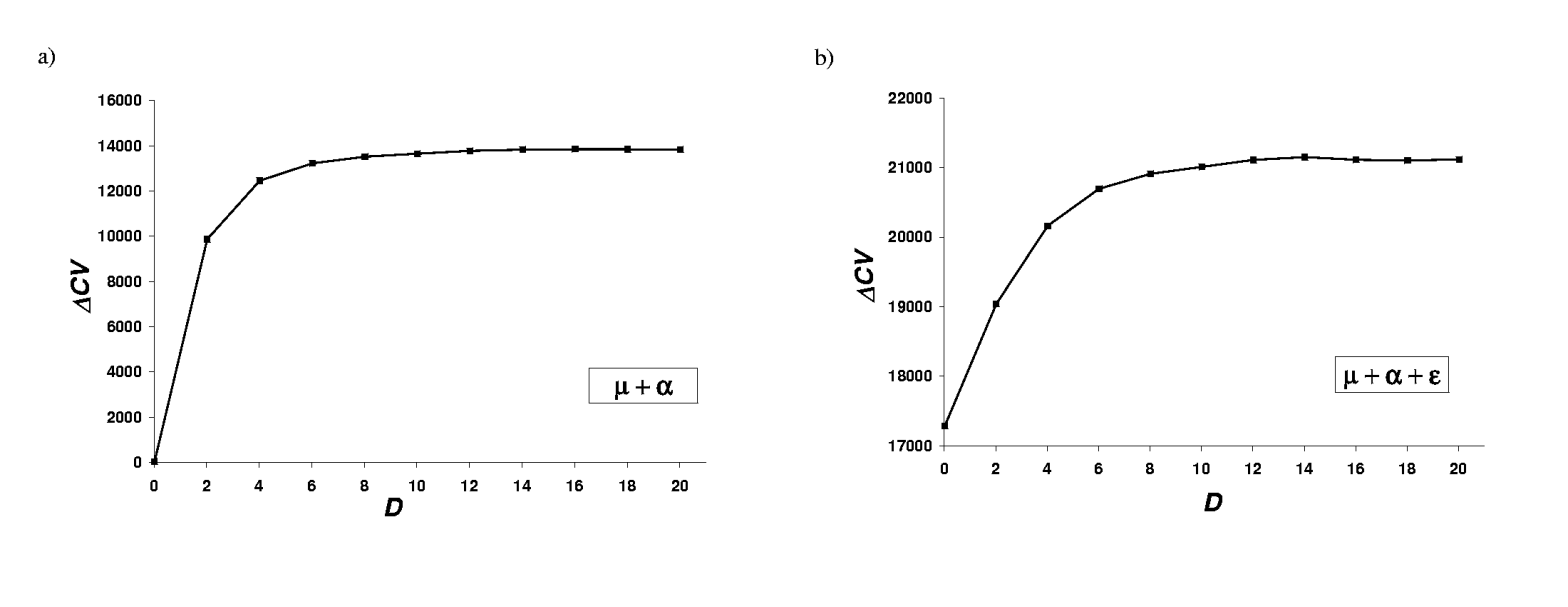
**Effect of the solvent accessibility definition on the potential**. Gain in cross-validation score (see Methods) as a function of the number of accessibility classes. The average gain for the 2-fold cross-validation experiment is shown. **(a) **Inverse potential consisting in solvent accessibility terms only, and **(b) **inverse potential combining contact and solvent accessibility terms.

The evolution of the CV score as a function of the number of accessibility classes (*D*) is shown in figure 3. When *D *increases, the fit of the model improves, until a point is reached where the penalization for model dimensionality starts to dominate the score. The optimal number of classes obtained is 14 to 16, depending on the form of the potential studied, although 4 to 6 classes is sufficient to attain 90% of the fit improvement.

**Figure 4:**
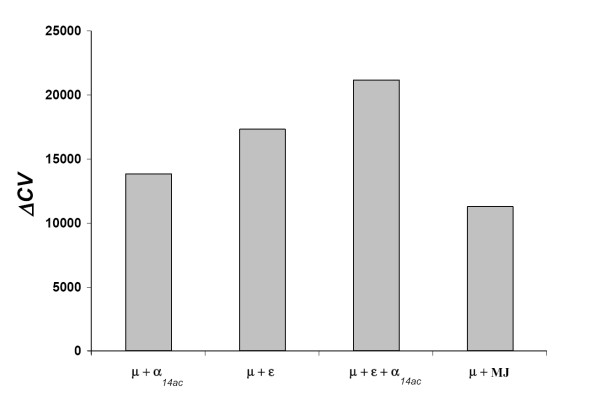
**Model comparison**. Cross-validation (CV) scores obtained for the different forms of potentials tested. The average gain (relative to the CV score obtained with the flat potential *μ*, see Methods) for the 2-fold cross-validation experiment is reported. *α*_14ac_: solvent accessibility potential, 14 accessibility classes; *ε*: contact potential; MJ: Miyazawa and Jernigan's potential.

The scores obtained for the different models tested are reported in figure 4. We also included in the comparison the Miyazawa and Jernigan potential [17]. The contact potential performs better than the pure solvent accessibility potential, and the combination of both terms is the most informative. Miyazawa and Jernigan's potential results in a poorer fit improvement than any of the other models.

### Specificity of the designed sequences

Once an optimal value of *θ *is obtained, properties of the sequences induced by the models can be investigated by sampling sequences from *p*(*s *| *c*, *θ*), using this optimal value of *θ*. In particular, we tested to what extent the sequences proposed by our method met the requirement of specificity, i.e. the condition that the sequences designed on a given conformation *c *indeed have *c *as their unique ground state. More precisely, we generated 20 sequences by Gibbs sampling for 60 randomly chosen structures [see Additional file 8], i.e. 1,200 sequences for each potential, and performed a fold recognition experiment for the designed sequences, monitoring the score for the target fold using THREADER [37] (figure 6 and Table 1).

The solvent accessibility potential alone (*α*_14ac _+ *μ*, figure 6b) is not sufficient to provide specificity to the designed sequences, and behaves almost as poorly as the flat potential (*μ*, figure 6a). A mild improvement is seen when using the contact potential (*ε *+ *μ*, figure 6c): for 10% of the designed sequences the target fold is found among the best scoring folds (Table 1), and the distribution of this ranking is skewed towards lower values. However, it is only with the combined potential (*ε *+ *μ*_14ac _+ *μ*, figure 6d) that a significant improvement is observed: for more than half of the designed sequences the target fold is found among the best 1% scoring folds, even though the average sequence identity with the native sequence is less than 10% in all cases (Table 1).

We also tested a subset of 120 randomly chosen designed sequences using another fold recognition program, LOOPP [38]. LOOPP is based on a combination of several structure prediction methods, based on threading, secondary structure, sequence profile and exposed surface area prediction. The results obtained with this program were similar to those of THREADER: for 51.2% of the designed sequences using the combined (*ε *+ *α*_14ac _+ *μ*) potential, the target fold was found as the first hit, and for 67.2% the target fold was found among the first 10 hits.

In contrast, many of the current fold recognition programs based on sequence profile methods produced no significant hits (data not shown), which is not surprising, given that our sampling algorithm produces highly divergent sequences, with no similarity to any natural protein.

## Discussion

The central idea of the present work is to reformulate the problem of devising statistical potentials for protein design as a statistical inference problem. This reformulation, based on the maximum likelihood (ML) principle, led us naturally to a gradient descent method, with the only additional aspect being that the gradient to follow is itself estimated by Monte-Carlo averaging.

The main advantage of this ML framework is that it guarantees an optimal predictive power of the resulting potential. In addition, it is very general, and can in principle be applied to any form of statistical potential. In particular, it is not restricted to coarse grained descriptions of proteins, and it could also be applied at the atomic level.

Interestingly, our gradient descent method turns out to be similar in spirit to an iterative scheme proposed by Thomas and Dill [39], although in that case the purpose was to optimize a potential in the context of the folding problem. Specifically, Thomas and Dill tune the potential so as to match the observed and expected number of contacts of each type, except that their expectation is taken on a set of alternative conformations, for a fixed sequence, whereas we take the expectation on a set of alternative sequences, on the conformation of interest. Note that Thomas and Dill derived their method from intuitive arguments, and not as a mathematical consequence of the ML principle.

These two alternative optimization schemes, obtained by normalizing either over the sequence or over the structure space, are quite distinct, at least conceptually. How the resulting potentials would differ in practice is more difficult to evaluate. Among other things, it will depend on how the approximation of *lnZ*_*s *_based on the random energy model works. In the eventuality that it does not work well, it is likely that the contact term of our inverse potential will in fact combine two things: the information corresponding to the conformational energy of the sequence itself, which is also encoded in classical potentials optimized for threading, plus some information coming from the decoy term ln *Z*_*s*_. A way to settle this question would be to optimize a contact potential using, on the same learning set, both normalization schemes, and then compare the resulting values as well as their predictive powers.

### Model assessment and comparison

The methodological framework proposed here offers reliable criteria for comparing the empirical fit of alternative models on real data. In this respect, it should be noted that the lack of a reliable objective criterion for evaluating different statistical potentials has often been invoked for justifying the use of on-lattice idealized models [23]. However, on-lattice approaches are only moderately interesting, as they completely ignore the problem of the robustness of the learning method to model violation. Coarse-grained statistical potentials are by definition over-simplified models of proteins, and therefore, model violation is an intrinsic feature of the protein design problem. In this respect, the statistical language is interesting, since it is still valid, even for fitting and assessing models that are known to be imperfect.

On the other hand, the intuitive idea underlying cross-validation, i.e. measuring the rate of prediction of the native sequence, is quite simple, and has been invoked and used several times previously [16,29,32,35,40]. What we propose here is a better formalization of this idea. Note that in contrast to previous methods, we do not measure the *marginal *native prediction rate at each site, but the *joint *probability of the native sequence. This can be important, as it accounts for possible correlations in the predictive distribution. For instance, two given positions may not display any particular pattern, when considered marginally, but may jointly follow charge or steric compensatory patterns. These phenomena will not be taken into account in the overall fit of the potential when measuring the marginal prediction rate, as is usually done. Technically speaking, the joint probability of the native sequence on the corresponding structure is extremely small, and cannot be evaluated just by counting the frequency at which the native sequence appears in the sample obtained by Gibbs sampling. For this, more elaborate numerical methods, such as thermodynamic integration, are required.

In the present case, the comparison between alternative models has allowed us to measure the relative contribution of each term of the potential and to refine the protein representation. The contact component turns out to be the most informative (figure 4), although it should be complemented with other energetic forms. Here, we have tested the addition of a solvent accessibility component, which significantly improves the fit of the model. Contact information and solvent exposure are correlated, which is reflected in the fact that the fit improvement of each term is not additive.

Our model comparison method also gives us a direct way of choosing the optimal number of solvent accessibility classes (figure 3). Here, we found a number of 14 to 16 classes, which is higher than what one may have expected and than what is usually used. Note that this number depends on the way the classes are defined; here, the classes are based on quantiles, but as an alternative, we also tried a linear definition (evenly splitting the whole range of accessibility surfaces into *D *bins), which gave us an even higher optimal number of classes (20 classes, data not shown). In general, the present methodology could be used to investigate different definitions of accessibility classes, to refine the pairwise contact definition, or any other elements of the structure representation included in the potential.

The fact that our potential has a significantly better predictive power than that of Miyazawa and Jernigan (MJ, figure 4) is trivially expected, by construction of the ML potential. What is more surprising is that the MJ matrix is less fit than a simple solvent-accessibility profile. A possible explanation would be that Miyazawa and Jernigan's potential is based on the quasi-chemical approximation, which is now known to be somewhat drastic [19,41,42], as it neglects correlations between observed pairing frequencies, due to chain connectivity and multiple contacts. Alternatively, it could mean that potentials optimized for folding are really not suited for protein design purposes. Testing other pairwise contact potentials, in particular those that do not rely on the quasi-chemical approximation [22,24,43-45], would be a way to address this issue.

### Sequence sampling

The method that we propose in this work is probabilistic in essence. As such, it offers a very natural framework for investigating the patterns induced by the models on distributions of sequences.

#### Specificity of the designed sequences

A sequence *s *designed for a target conformation *c *should not only be compatible with *c*, but also incompatible with competing folds. A rigorous solution to this problem involves a simultaneous search over the sequence and conformation space. It is possible, however, to achieve specificity without explicitly seeking to penalize competing states (*negative *design), if we rely on the approximation based on the random energy model, where the normalization constant of equation 4 can be considered as a function of the sequence composition only [25,46]. In our case, the normalization of the likelihood will also play an important role: since the total probability over all possible sequences has to be 1, maximizing the probability for a given sequence *s*_1 _on its native conformation *c*_1 _will lower the probability that another natural sequence *s*_2_, with native conformation *c*_2_, also gets a high probability on *c*_1_. When many sequences are learnt in parallel, this phenomenon should ultimately favor specificity of *s*_2 _on *c*_2_, compared to all other conformations of the data set.

On the other hand, the extent to which the specificity is achieved will depend on the actual form of the potential used, as well as on the data base used for learning. To address this question, we produced a large number of sequences with four different potentials, and checked their ability to recognize the target fold, as measured by the *Z*-score ratio or by the ranking of the target structure in a fold recognition experiment. Indeed, an improvement of specificity is observed when using better potentials, suggesting that the method is effectively capturing specific dependencies between the conformation and the sequence of the proteins in the learning set, even for the simple forms of potentials tested here. For the combined (*ε *+ *α*_14ac _+ *μ*) potential, the average *Z*-score ratio of the designed sequences is similar to what has been reported for other protein design algorithms [46]. Conversely, this also suggests that a more sophisticated potential may further improve the specificity of the sequences designed using our algorithm.

#### Conformation-dependent site-specific profiles

**Figure 5:**
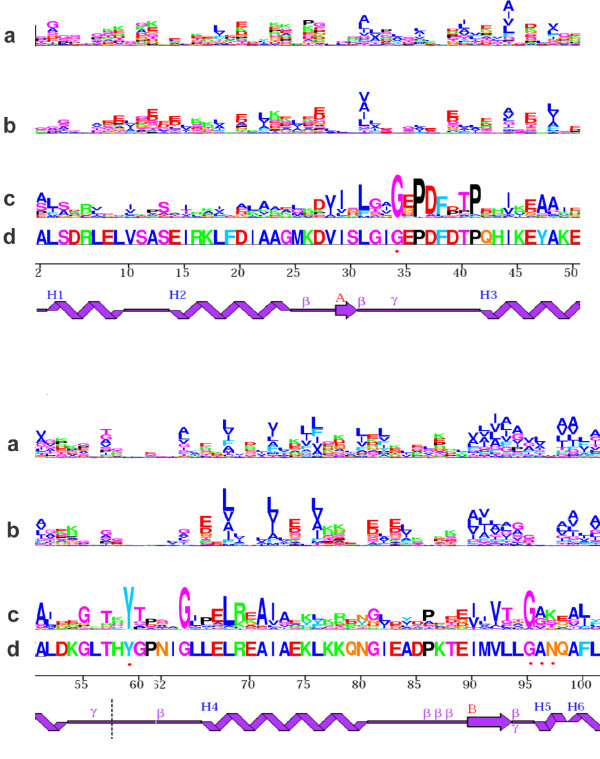
**Site-specific profiles**. Sequence logos of site-specific profiles induced on an alpha-aminotransferase ([PDB:1GDE], chain A), using a contact + solvent accessibility (14 classes) potential. From top to bottom: **(a) **marginal profiles, **(b) **leave-one-out profiles, **(c) **empirical profiles from a multiple sequence alignment of 162 sequences [see Additional file 4], and **(d) **native sequence of the reference protein. Secondary structure representation was taken from PDBsum [57]. Red dot: residue interaction with ligand. Only the first 100 amino acids are shown; sequence logos for the whole protein are available as supplementary material [see Additional file 5] [see Additional file 6].

**Figure 6:**
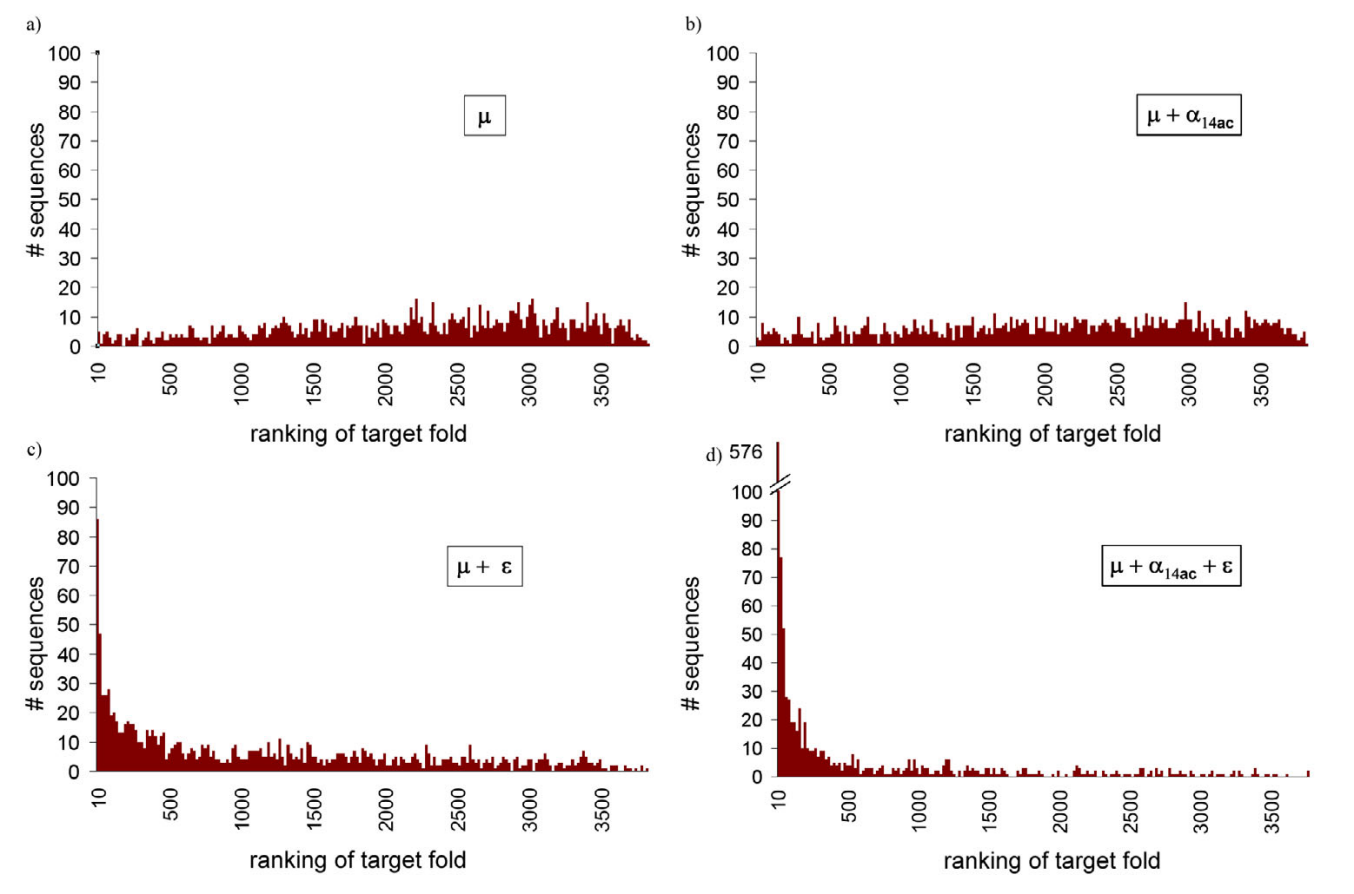
**Design specificity**. Histograms of the ranking of the target structure in a fold recognition experiment using THREADER. 20 sequences were generate for 60 randomly chosen structures, using (a) a flat (*μ*) potential, (b) a solvent accessibility, 14 classes (*μ *+ *α*_14ac_) potential, (c) a contact (*μ *+ *ε*) potential, and (d) the combined (*μ *+ *α*_14ac _+ *ε*) potential.

To compare natural protein sequences with those predicted by the optimized potentials, marginal, leave-one-out and empirical profiles (see methods) were generated for the 60 proteins used in the design specificity experiment described above; the profiles obtained for the best and the worst scoring structures are provided as supplementary materials [see Additional file 7]. Overall, leave-one-out profiles (figure 5a) and marginal profiles (figure 5b) do not display significant differences in the discriminative power between sites: the mean Shannon entropy per site is 0.743 ± 0.366 for marginal profiles, and 0.696 ± 0.428 for leave-one-out profiles. It is worth noting that the mean entropy per site for each protein, and the corresponding standard deviation, i.e. the average amount of information at each site and the variation between sites, are both correlated with the performance of the particular protein in the fold recognition experiment, and this, only for the combined (*ε *+ *α*_14ac _+ *μ*) potential (Table 1).

A detailed analysis of the leave-one-out profiles for a particular case, an alpha-aminotransferase, may be useful to understand which type of information is effectively captured by the potential, and which is not captured at all, thereby suggesting possible ways of improving the current form of potential.

First, regions of the protein that show little secondary structure (such as in positions 32–40, 55–65 and 82–88) contain less information (mean entropy per site = 0.756) than regions with local structure (mean entropy per site = 0.856). This is not surprising, since these regions typically have fewer contacts between residues, and thus the amount of information included in the protein representation is lower.

Concerning regions with defined secondary structure, residue polarity is the information most easily captured. Charged residues are also distinctively inferred, as well as glycines, to a lesser extent (e.g. glycine 64, 81 and 95 – the latter predicted at position 94 or 95). In contrast, prolines are rarely correctly predicted, which is expected, since the properties most distinctive of prolines (such as phi-psi dihedral angles or local secondary structure) are not included in this particular form of potential.

Interestingly, some residues that have a crucial importance for the protein structure or function fail to be predicted, simply because the properties conferring their importance are not included in the protein description. This is the case of the amino acids that are in close interaction with a ligand (positions 34, 59, 96, 97).

Finally, the leave-one-out profiles display an interesting behavior with respect to positions where the amino-acid present in the reference sequence is not at all conserved in other members of the family. In some cases, they simply do not predict anything (e.g. glycines 24 and 60, or leucine 9, isoleucine 21, and alanine 23), which suggests that their limited importance in structure stability or function is recognized by the inverse potential. In other cases, the natural profile is even reproduced in the leave-one-out profile, instead of the amino acid of the reference sequence; such is the case for phenylalanine 100.

## Conclusion

As illustrated by the sequence logos and the fold recognition experiments performed above, the predictive power of the models proposed here is encouraging, but nevertheless still weak. It is not yet clear to what extent this is due to the specific choice made concerning the form of the statistical potential, to the approximation of ln *Z*_*s *_as a function of the sole composition of the sequence, or to yet other reasons. Most probably, we are facing a combination of several factors. The methods proposed here can now be used to address these difficult questions empirically.

In one direction, other approximations of ln *Z*_*s*_, less drastic than the random energy model, but still accessible in practice, can be investigated. For instance, following Deutsch and Kurozky (1996), the conditional probability of a sequence could be defined as:

*p*(*s *| *c*) ∝ *e*^-[*E*(*S*,*C*)-〈*E*(*S*)〉] ^*p*(*s*)       (37)

where the expectation 〈·〉 is taken over a pre-defined set of decoy conformations. More sophisticated Monte Carlo methods, jointly sampling the sequence and conformation spaces, can also be imagined, in order to get more precise evaluations of ln *Z*_*s*_, while staying in the same global maximum likelihood formalism.

On the other hand, all the many statistical potentials that have been proposed over the last fifteen years may in principle be investigated in the same way as we have done here. In particular, distance-dependent potentials [47] and main-chain dihedral angle potentials [48], which imply a richer representation of the protein structure, may result in models of greater predictive power. Other ways of implicitly considering side-chain conformation may also be easily incorporated into the model.

In a completely different perspective, it is possible to devise probabilistic models that are not exclusively defined in terms of a conformational free energy, even in a formal way. For instance, additional terms, concerning secondary structure aspects, interactions between successive positions along the sequence, or terms related to the folding constraints, can all be combined in an additive manner in the inverse potential. In fact, the model need not even be formulated in terms of a Boltzmann distribution, as long as the parameters are fitted by ML, and the predictive power of the resulting models is evaluated in a systematic way. Altogether, this amounts to setting up a robust statistical framework helping us to understand how, and to what extent, the sequences of natural proteins are determined by protein structure.

## Methods

### Structure representation

We used Miyazawa and Jernigan's definition of contacts [17]: each residue is represented by the center of its side chain atom positions; the positions of C^*α *^atoms are used for glycine. Residues whose centers are closer than 6.5Å are defined to be in contact. The accessible surface of a residue is defined as the atomic accessible area when a probe of the radius of a molecule of water is rolled around the Van der Waal's surface of the protein [49]. We used the program Naccess [50] to make this calculation. When treating PDB files with multiple chains, solvent accessibility was calculated taking into account all molecules in the structure. The accessibility classes (percentage relative to the accessibility in Ala-X-Ala fully extended tripeptide) were defined so as to generate *D *equal-sized subsets of sites. The complete definition of accessibility classes is available as supporting material [see Additional file 1].

### Monte Carlo implementation

In order to calculate the derivative of *ω *in the gradient descent procedure, expectations with respect to *p*(*S*' | *C*, *θ*)in equation 33 are evaluated numerically. A sample  drawn from *p*(*S *| *C*, *θ*) is obtained by a Gibbs sampling algorithm similar to that of Robinson et al. [10]. The elementary cycle of our Gibbs sampler is as follows: for each *p *= 1..*P*, and for each *i *= 1..*N*_*P*_, each of the 20 amino acids is proposed at site *i *of protein *p*, by successively setting sip
 MathType@MTEF@5@5@+=feaafiart1ev1aaatCvAUfKttLearuWrP9MDH5MBPbIqV92AaeXatLxBI9gBaebbnrfifHhDYfgasaacH8akY=wiFfYdH8Gipec8Eeeu0xXdbba9frFj0=OqFfea0dXdd9vqai=hGuQ8kuc9pgc9s8qqaq=dirpe0xb9q8qiLsFr0=vr0=vr0dc8meaabaqaciaacaGaaeqabaqabeGadaaakeaacqWGZbWCdaqhaaWcbaGaemyAaKgabaGaemiCaahaaaaa@310C@ = *a*, for all *a *= 1..20; in each case, the energy change Δ*G*_*a *_induced by this point substitution is evaluated; then, sip
 MathType@MTEF@5@5@+=feaafiart1ev1aaatCvAUfKttLearuWrP9MDH5MBPbIqV92AaeXatLxBI9gBaebbnrfifHhDYfgasaacH8akY=wiFfYdH8Gipec8Eeeu0xXdbba9frFj0=OqFfea0dXdd9vqai=hGuQ8kuc9pgc9s8qqaq=dirpe0xb9q8qiLsFr0=vr0=vr0dc8meaabaqaciaacaGaaeqabaqabeGadaaakeaacqWGZbWCdaqhaaWcbaGaemyAaKgabaGaemiCaahaaaaa@310C@ is set to amino acid *a *with probability pa∝e−ΔGa.
 MathType@MTEF@5@5@+=feaafiart1ev1aaatCvAUfKttLearuWrP9MDH5MBPbIqV92AaeXatLxBI9gBaebbnrfifHhDYfgasaacH8akY=wiFfYdH8Gipec8Eeeu0xXdbba9frFj0=OqFfea0dXdd9vqai=hGuQ8kuc9pgc9s8qqaq=dirpe0xb9q8qiLsFr0=vr0=vr0dc8meaabaqaciaacaGaaeqabaqabeGadaaakeaacqWGWbaCdaWgaaWcbaGaemyyaegabeaakiabg2Hi1kabdwgaLnaaCaaaleqabaGaeyOeI0IaeuiLdqKaem4raC0aaSbaaWqaaiabdggaHbqabaaaaOGaeiOla4caaa@3866@. After *Q *cycles of burnin, a series of *h *= 1..*K*_*EM *_cycles are performed, and after each cycle, the current sequence, *S*_*h*_, is recorded. Once the sample is obtained, the expectation (32) is evaluated as

〈∂G∂θ〉≃1KEM∑h=1KEM∂G(Sh,C)∂θ     (38)
 MathType@MTEF@5@5@+=feaafiart1ev1aaatCvAUfKttLearuWrP9MDH5MBPbIqV92AaeXatLxBI9gBaebbnrfifHhDYfgasaacH8akY=wiFfYdH8Gipec8Eeeu0xXdbba9frFj0=OqFfea0dXdd9vqai=hGuQ8kuc9pgc9s8qqaq=dirpe0xb9q8qiLsFr0=vr0=vr0dc8meaabaqaciaacaGaaeqabaqabeGadaaakeaacqGHPms4daWcaaqaaGGaciab=jGi2kabdEeahbqaaiab=jGi2kab=H7aXbaacqGHQms8cqWIdjYodaWcaaqaaiabigdaXaqaaiabdUealnaaBaaaleaacqWGfbqrcqWGnbqtaeqaaaaakmaaqahabaWaaSaaaeaacqWFciITcqWGhbWrdaqadaqaaiabdofatnaaBaaaleaacqWGObaAaeqaaOGaeiilaWIaem4qameacaGLOaGaayzkaaaabaGae8NaIyRae8hUdehaaaWcbaGaemiAaGMaeyypa0JaeGymaedabaGaem4saS0aaSbaaWqaaiabdweafjabd2eanbqabaaaniabggHiLdGccaWLjaGaaCzcamaabmaabaGaeG4mamJaeGioaGdacaGLOaGaayzkaaaaaa@5535@

and the derivative of *ω *with respect to *θ *follows immediately.

The overall gradient descent procedure runs as follows: we start from a random potential *θ*_0 _and a random set of sequences, and perform the following iterative scheme:

• perform *Q *Gibbs cycles for the burnin, and *k*_*EM *_additional cycles for the sampling itself. Keep the final sequences as the starting point of the next cycle.

• update *θ *by gradient descent, based on the estimate of the gradient obtained over the sample:

θn+1=θn−δθ.∂ω(S)∂θ     (39)
 MathType@MTEF@5@5@+=feaafiart1ev1aaatCvAUfKttLearuWrP9MDH5MBPbIqV92AaeXatLxBI9gBaebbnrfifHhDYfgasaacH8akY=wiFfYdH8Gipec8Eeeu0xXdbba9frFj0=OqFfea0dXdd9vqai=hGuQ8kuc9pgc9s8qqaq=dirpe0xb9q8qiLsFr0=vr0=vr0dc8meaabaqaciaacaGaaeqabaqabeGadaaakeaaiiGacqWF4oqCdaWgaaWcbaGaemOBa4Maey4kaSIaeGymaedabeaakiabg2da9iab=H7aXnaaBaaaleaacqWGUbGBaeqaaOGaeyOeI0Iae8hTdqMae8hUdeNaeiOla4YaaSaaaeaacqWFciITcqWFjpWDdaqadaqaaiabdofatbGaayjkaiaawMcaaaqaaiab=jGi2kab=H7aXbaacaWLjaGaaCzcamaabmaabaGaeG4mamJaeGyoaKdacaGLOaGaayzkaaaaaa@490A@

where . is a scalar product, and *δθ *is a step-vector. In practice, the coefficients of *δθ *are tuned empirically, allowing three degrees of freedom, for the *α*, the *ε*, and the *μ *component of the potential respectively.

• iterate.

As a stopping rule, we monitor the evolution of *ω*(*θ*) itself, which we evaluate every 100 steps by a numerical procedure (see below), and stop when *ω*(*θ*) has stabilized. In practice, we used *Q *= 100 and *k*_*EM *_= 100. At first sight, it would seem that a larger number of points *k*_*EM *_would be needed to get a precise expectation, but in the present case one can rely on the self-averaging of the derivatives across the 100,000 sites of the database.

### Likelihood evaluation

The difficult part in estimating the likelihood (or equivalently *ω*(*θ*)), for a given value of *θ*, is to obtain an evaluation of ln *Y*. We do this by thermodynamic integration, or path sampling [51,52], using the quasi-static method which we developed previously [53].

First, for 0 ≤ *β *≤ 1, we define



The associated probability distribution is:

pβ(s|c,θ)=e−Gβ(s,c)Yβ,     (41)
 MathType@MTEF@5@5@+=feaafiart1ev1aaatCvAUfKttLearuWrP9MDH5MBPbIqV92AaeXatLxBI9gBaebbnrfifHhDYfgasaacH8akY=wiFfYdH8Gipec8Eeeu0xXdbba9frFj0=OqFfea0dXdd9vqai=hGuQ8kuc9pgc9s8qqaq=dirpe0xb9q8qiLsFr0=vr0=vr0dc8meaabaqaciaacaGaaeqabaqabeGadaaakeaacqWGWbaCdaWgaaWcbaacciGae8NSdigabeaakmaabmaabaGaem4CamNaeiiFaWNaem4yamMaeiilaWIae8hUdehacaGLOaGaayzkaaGaeyypa0ZaaSaaaeaacqWGLbqzdaahaaWcbeqaaiabgkHiTiabdEeahnaaBaaameaacqWFYoGyaeqaaSWaaeWaaeaacqWGZbWCcqGGSaalcqWGJbWyaiaawIcacaGLPaaaaaaakeaacqWGzbqwdaWgaaWcbaGae8NSdigabeaaaaGccqGGSaalcaWLjaGaaCzcamaabmaabaGaeGinaqJaeGymaedacaGLOaGaayzkaaaaaa@4C8A@

Yβ=∑s′e−Gβ(s′,c).     (42)
 MathType@MTEF@5@5@+=feaafiart1ev1aaatCvAUfKttLearuWrP9MDH5MBPbIqV92AaeXatLxBI9gBaebbnrfifHhDYfgasaacH8akY=wiFfYdH8Gipec8Eeeu0xXdbba9frFj0=OqFfea0dXdd9vqai=hGuQ8kuc9pgc9s8qqaq=dirpe0xb9q8qiLsFr0=vr0=vr0dc8meaabaqaciaacaGaaeqabaqabeGadaaakeaacqWGzbqwdaWgaaWcbaacciGae8NSdigabeaakiabg2da9maaqafabaGaemyzau2aaWbaaSqabeaacqGHsislcqWGhbWrdaWgaaadbaGae8NSdigabeaalmaabmaabaGafm4CamNbauaacqGGSaalcqWGJbWyaiaawIcacaGLPaaaaaaabaGafm4CamNbauaaaeqaniabggHiLdGccqGGUaGlcaWLjaGaaCzcamaabmaabaGaeGinaqJaeGOmaidacaGLOaGaayzkaaaaaa@448C@

What we are looking for is In *Y*_1_. As for In *Y*_0_, it factors out, and can be computed directly:

ln⁡Y0=Nln⁡(∑a=120e−μa).     (43)
 MathType@MTEF@5@5@+=feaafiart1ev1aaatCvAUfKttLearuWrP9MDH5MBPbIqV92AaeXatLxBI9gBaebbnrfifHhDYfgasaacH8akY=wiFfYdH8Gipec8Eeeu0xXdbba9frFj0=OqFfea0dXdd9vqai=hGuQ8kuc9pgc9s8qqaq=dirpe0xb9q8qiLsFr0=vr0=vr0dc8meaabaqaciaacaGaaeqabaqabeGadaaakeaacyGGSbaBcqGGUbGBcqWGzbqwdaWgaaWcbaGaeGimaadabeaakiabg2da9iabd6eaojGbcYgaSjabc6gaUnaabmaabaWaaabCaeaacqWGLbqzdaahaaWcbeqaaiabgkHiTGGaciab=X7aTnaaBaaameaaieGacqGFHbqyaeqaaaaaaSqaaiabdggaHjabg2da9iabigdaXaqaaiabikdaYiabicdaWaqdcqGHris5aaGccaGLOaGaayzkaaGaeiOla4IaaCzcaiaaxMaadaqadaqaaiabisda0iabiodaZaGaayjkaiaawMcaaaaa@4AFB@

We can thus equivalently evaluate the difference ln *Y*_1 _– ln *Y*_0_. To do this, we rely on the following identity:

ln⁡Y1−ln⁡Y0=∫01∂ln⁡Y∂βdβ(44)=∫01〈∂G∂β〉βdβ,(45)
 MathType@MTEF@5@5@+=feaafiart1ev1aaatCvAUfKttLearuWrP9MDH5MBPbIqV92AaeXatLxBI9gBaebbnrfifHhDYfgasaacH8akY=wiFfYdH8Gipec8Eeeu0xXdbba9frFj0=OqFfea0dXdd9vqai=hGuQ8kuc9pgc9s8qqaq=dirpe0xb9q8qiLsFr0=vr0=vr0dc8meaabaqaciaacaGaaeqabaqabeGadaaakeaafaqadeGacaaabaGagiiBaWMaeiOBa4MaemywaK1aaSbaaSqaaiabigdaXaqabaGccqGHsislcyGGSbaBcqGGUbGBcqWGzbqwdaWgaaWcbaGaeGimaadabeaakiabg2da9maapedabaWaaSaaaeaaiiGacqWFciITcyGGSbaBcqGGUbGBcqWGzbqwaeaacqWFciITcqWFYoGyaaaaleaacqaIWaamaeaacqaIXaqma0Gaey4kIipakiabdsgaKjab=j7aIbqaamaabmaabaGaeeinaqJaeeinaqdacaGLOaGaayzkaaaabaGaeyypa0Zaa8qmaeaacqGHPms4daWcaaqaaiab=jGi2kabdEeahbqaaiab=jGi2kab=j7aIbaacqGHQms8daWgaaWcbaGae8NSdigabeaaaeaacqaIWaamaeaacqaIXaqma0Gaey4kIipakiabdsgaKjab=j7aIjabcYcaSaqaamaabmaabaGaeGinaqJaeGynaudacaGLOaGaayzkaaaaaaaa@62F5@

where 〈·〉_*β *_is the expectation over *p*_*β*_(*s*' | *c*, *θ*).

In practice, the method consists in first equilibrating the Gibbs sampler at *β *= 0, and then, performing a series of *K*_*Th *_+ 1 cycles, where at each step, the value of *β *is increased by a small amount *δβ *= 1/*K*_*Th*_. The successive values of ∂G∂β
 MathType@MTEF@5@5@+=feaafiart1ev1aaatCvAUfKttLearuWrP9MDH5MBPbIqV92AaeXatLxBI9gBaebbnrfifHhDYfgasaacH8akY=wiFfYdH8Gipec8Eeeu0xXdbba9frFj0=OqFfea0dXdd9vqai=hGuQ8kuc9pgc9s8qqaq=dirpe0xb9q8qiLsFr0=vr0=vr0dc8meaabaqaciaacaGaaeqabaqabeGadaaakeaadaWcaaqaaGGaciab=jGi2kabdEeahbqaaiab=jGi2kab=j7aIbaaaaa@3239@ obtained during this quasi-static sampling scheme are recorded, and their average is our estimate of ln *Y*_1 _– ln *Y*_0_:



Note that these developments are for one protein, but the generalization over the database is straightforward.

In the conditions of the present work, *K*_*Th *_= 1, 000 is sufficient to obtain an estimate of ln *Y*_1 _– ln *Y*_0 _with an error less than one natural unit of logarithm.

### Model comparison

We measured the fit of each model using cross-validation (CV): the potentials optimized on a first data set, i.e. the learning set, (*θ*_*L*_) are applied on the second data set (the test set), and the log-likelihood is directly taken as a measure of fit. More precisely, for each model *M*,

*CV*_*M *_= -ln *p*(*S*_*T *_| *C*_*T*_, *θ*_*L*_, *M*),      (47)

where *S*_*T *_and *C*_*T *_are the sequences and structures of the test set. The difference with the CV score obtained for the flat potential (*μ*) is reported: Δ*CV *= *CV*_*μ *_– *CV*_*M*_.

### Sequence sampling: site-specific profiles

Once an optimal value of *θ *is obtained, sequences compatible with a given conformation can be sampled from *p*(*s *| *c*, θ^
 MathType@MTEF@5@5@+=feaafiart1ev1aaatCvAUfKttLearuWrP9MDH5MBPbIqV92AaeXatLxBI9gBaebbnrfifHhDYfgasaacH8akY=wiFfYdH8Gipec8Eeeu0xXdbba9frFj0=OqFfea0dXdd9vqai=hGuQ8kuc9pgc9s8qqaq=dirpe0xb9q8qiLsFr0=vr0=vr0dc8meaabaqaciaacaGaaeqabaqabeGadaaakeaadaqiaaqaaGGaciab=H7aXbGaayPadaaaaa@2F2B@) by Gibbs sampling, and then further investigated. For instance, the frequency of each of the 20 amino acids (*a*) at each position (*i*) can be computed (*q*_*i*_(*a*)), yielding a vector of site-specific *marginal *profiles, graphically displayed as sequence logos [54]. Alternatively, *leave-one-out *profiles can be obtained by computing the probability of each of the 20 amino-acids at each site of the test sequence, given the potential and the native sequence at all other positions:

*p*(*s*_*i *_= *a *| *s*_*j*_, *j *≠ *i*, *θ*).      (48)

We measured the amount of information displayed by the profiles using the site-specific Shannon entropy:

hi=−∑aqi(a)ln⁡qi(a)     (49)
 MathType@MTEF@5@5@+=feaafiart1ev1aaatCvAUfKttLearuWrP9MDH5MBPbIqV92AaeXatLxBI9gBaebbnrfifHhDYfgasaacH8akY=wiFfYdH8Gipec8Eeeu0xXdbba9frFj0=OqFfea0dXdd9vqai=hGuQ8kuc9pgc9s8qqaq=dirpe0xb9q8qiLsFr0=vr0=vr0dc8meaabaqaciaacaGaaeqabaqabeGadaaakeaacqWGObaAdaWgaaWcbaGaemyAaKgabeaakiabg2da9iabgkHiTmaaqafabaGaemyCae3aaSbaaSqaaiabdMgaPbqabaGcdaqadaqaaiabdggaHbGaayjkaiaawMcaaaWcbaGaemyyaegabeqdcqGHris5aOGagiiBaWMaeiOBa4MaemyCae3aaSbaaSqaaiabdMgaPbqabaGcdaqadaqaaiabdggaHbGaayjkaiaawMcaaiaaxMaacaWLjaWaaeWaaeaacqaI0aancqaI5aqoaiaawIcacaGLPaaaaaa@482A@

We compared both marginal and leave-one-out profiles to the *empirical *profiles, i.e. profiles displayed by natural sequences. We generated these empirical profiles from multiple sequence alignments obtained from the ConSurf-HSSP database [55].

### Sequence sampling: design specificity

As a test for specificity, designed sequences were submitted to a fold recognition experiment, using the fold recognition program THREADER [37]. In THREADER, the compatibility of a sequence *s *for a given structure *c *is measured by the *Z*-score:

Z=〈E(s,C)〉−E(s,c)σ     (50)
 MathType@MTEF@5@5@+=feaafiart1ev1aaatCvAUfKttLearuWrP9MDH5MBPbIqV92AaeXatLxBI9gBaebbnrfifHhDYfgasaacH8akY=wiFfYdH8Gipec8Eeeu0xXdbba9frFj0=OqFfea0dXdd9vqai=hGuQ8kuc9pgc9s8qqaq=dirpe0xb9q8qiLsFr0=vr0=vr0dc8meaabaqaciaacaGaaeqabaqabeGadaaakeaacqWGAbGwcqGH9aqpdaWcaaqaaiabgMYiHlabdweafnaabmaabaGaem4CamNaeiilaWIaem4qameacaGLOaGaayzkaaGaeyOkJeVaeyOeI0Iaemyrau0aaeWaaeaacqWGZbWCcqGGSaalcqWGJbWyaiaawIcacaGLPaaaaeaaiiGacqWFdpWCaaGaaCzcaiaaxMaadaqadaqaaiabiwda1iabicdaWaGaayjkaiaawMcaaaaa@4620@

where 〈*E*(*S*, *C*)〉 is the average of the THREADER statistical potential over all conformations of the decoy set, and *σ *is the corresponding standard deviation.

We randomly chose 70 structures of sizes ranging from 100 to 300 residues from the default THREADER dataset [see Additional file 8]. Structures whose native sequences produced a *Z*-score < 3 were discarded for the analysis. For each structure, *c*, we sampled 20 sequences from *p*(*s *| *c*, θ^
 MathType@MTEF@5@5@+=feaafiart1ev1aaatCvAUfKttLearuWrP9MDH5MBPbIqV92AaeXatLxBI9gBaebbnrfifHhDYfgasaacH8akY=wiFfYdH8Gipec8Eeeu0xXdbba9frFj0=OqFfea0dXdd9vqai=hGuQ8kuc9pgc9s8qqaq=dirpe0xb9q8qiLsFr0=vr0=vr0dc8meaabaqaciaacaGaaeqabaqabeGadaaakeaadaqiaaqaaGGaciab=H7aXbGaayPadaaaaa@2F2B@) by Gibbs sampling. These designed sequences were then submitted to THREADER [37], and their specificity for the target structure *c *was measured by the ranking of *c *among all other structures, sorted by increasing *Z*-score.

A subset of 120 among the 1,200 sequences generated with the combined (*ε *+ *α*_14ac _+ *μ*) potential (3–5 sequences for 23 distinct conformations, chosen at random; [see Additional file 8]) were also submitted to another fold recognition program, LOOPP [38], and the presence of the native conformation *c *as the first hit or in the first 10 hits was recorded.

### Learning databases

We used proteins culled from the entire PDB according to structure quality (resolution better than 2.0 Å) and with less than 25% of mutual sequence identity [56]. Two subsets of approximately equal size were obtained by partitioning the proteins randomly: DS1, 449 proteins, 100,077 sites, and DS2, 465 proteins, 99,894 sites. The final list of proteins is available as supporting material [see Additional file 2] [see Additional file 3].

## Authors' contributions

CLK participated in the implementation of the methods, performed the run of all the experiments, and co-wrote the manuscript. NR participated in the implementation of the methods, extensively supervised CLK and CB, and made contributions to the drafting of the manuscript. CB participated in the initial implementation of the methods. HP contributed to the drafting of the manuscript and the coordination of the project. NL set up the theoretical framework, co-wrote the manuscript, participated in the implementation of the methods and directed the overall project. All authors read and approved the final manuscript.

## Supplementary Material

Additional file 1 Extensive definition of accessibility classes Click here for file

Additional file 2 Data set DS1 – List of PDB identifiers of proteins used Click here for file

Additional file 3 Data set DS2 – List of PDB identifiers of proteins used Click here for file

Additional file 4 Multiple sequence alignment for sequence logos of figure 5. Multiple sequence alignment (Clustal format) used to generate sequence logos of figure 5. Click here for file

Additional file 5 Marginal and leave-one-out profiles of complete protein partially displayed in figure 5 Click here for file

Additional file 6 Empirical profiles of complete protein partially displayed in figure 5 Click here for file

Additional file 7 Marginal and leave-one-out profiles of 10 proteins used in the design specificity experiment Click here for file

Additional file 8 Table 1: list of PDB identifiers of proteins used in the design specificity experiment, and scores obtained for each one of the proteins, using the combined (*ε *+ *α*_14ac _+ *μ*) potential Click here for file

## Figures and Tables

**Table 1:** Specificity of designed sequences.

Potential	Average Z-score ratio	SDev Z-score ratio	Ranking (median)	Target fold in top 1% (A)	Target fold in top 10%	Average seq. identity	Correlation between (A) and mean entropy/site
*μ*	-0.12	0.18	2249	0.5%	4.8%	5.76 %	-0.26
*μ *+ *α*_14ac_	-0.10	0.18	2090	0.4%	6.3%	6.65 %	-0.04
*μ *+ *ε*	0.13	0.16	816.8	10.7%	33.5%	6.69 %	0.23
*μ *+ *α*_14ac _+ *ε*	0.45	0.23	32.7	53.6%	77.5%	7.82 %	0.64

Scores of a fold recognition experiment for designed sequences (see Methods). 1,200 sequences were sampled from *p*(*s *| *c*, *θ*) for each potential, and submitted to THREADER for fold recognition. Z-score ratio: Z-score of designed sequence/Z-score of native sequence in target fold.
