# Supplementary material for: A maximum likelihood framework for protein design
Source: BMC Bioinformatics. 2006 Jun 29;7:326. doi: 10.1186/1471-2105-7-326 (PMC1570151; doi:10.1186/1471-2105-7-326)
Supplement: Additional file 5 — Marginal and leave-one-out profiles of complete protein partially displayed in figure 5 [file 1471-2105-7-326-S5.pdf]

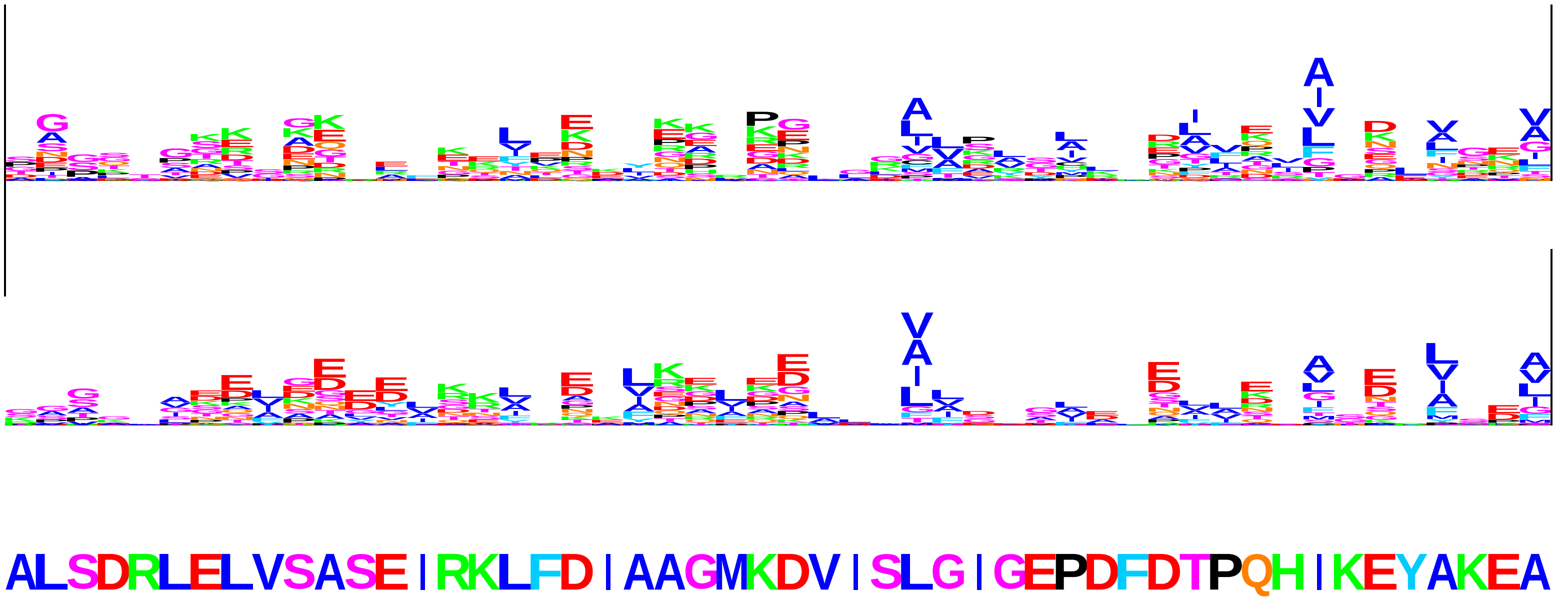
ALSDRLELVSASEIRKLFDIAAGMKDVISLGIGEPDFDTPQHIKEYAKEA

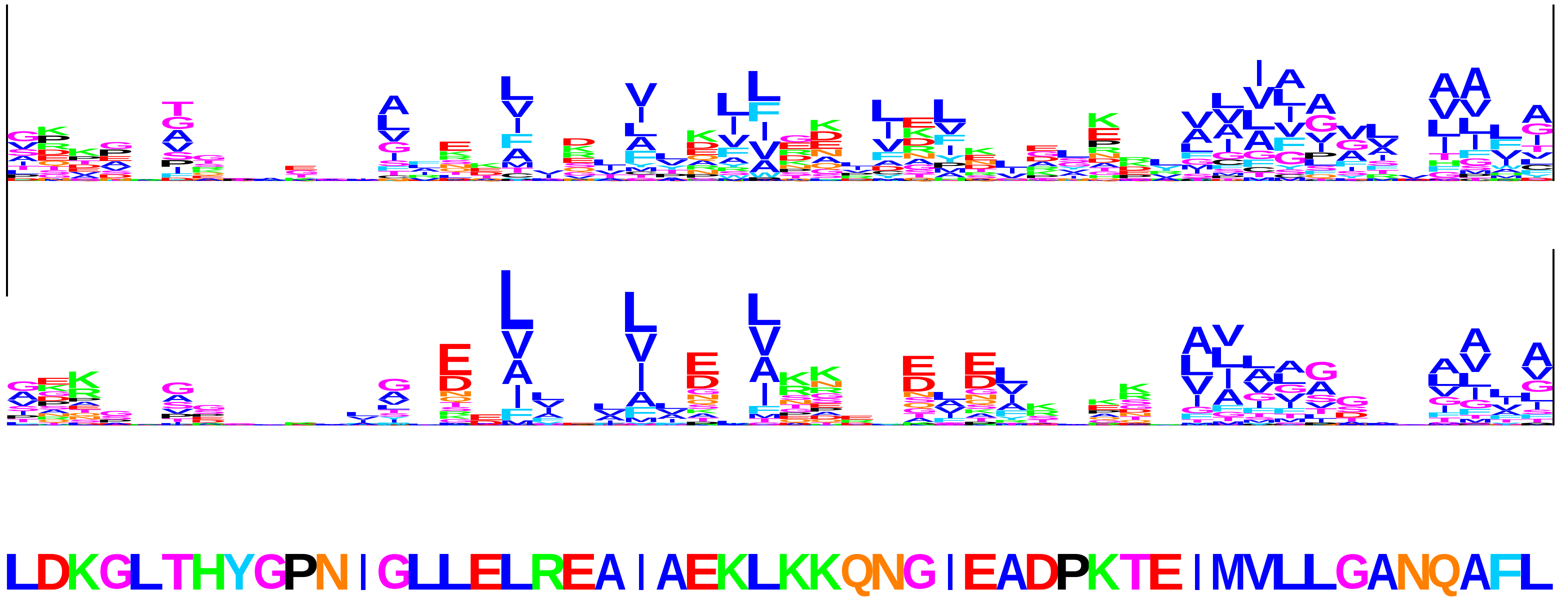
LDKGLTHYGPNIGLLELREAIAEKLKKQNGIEADPKTEIMVLLGANQAFL

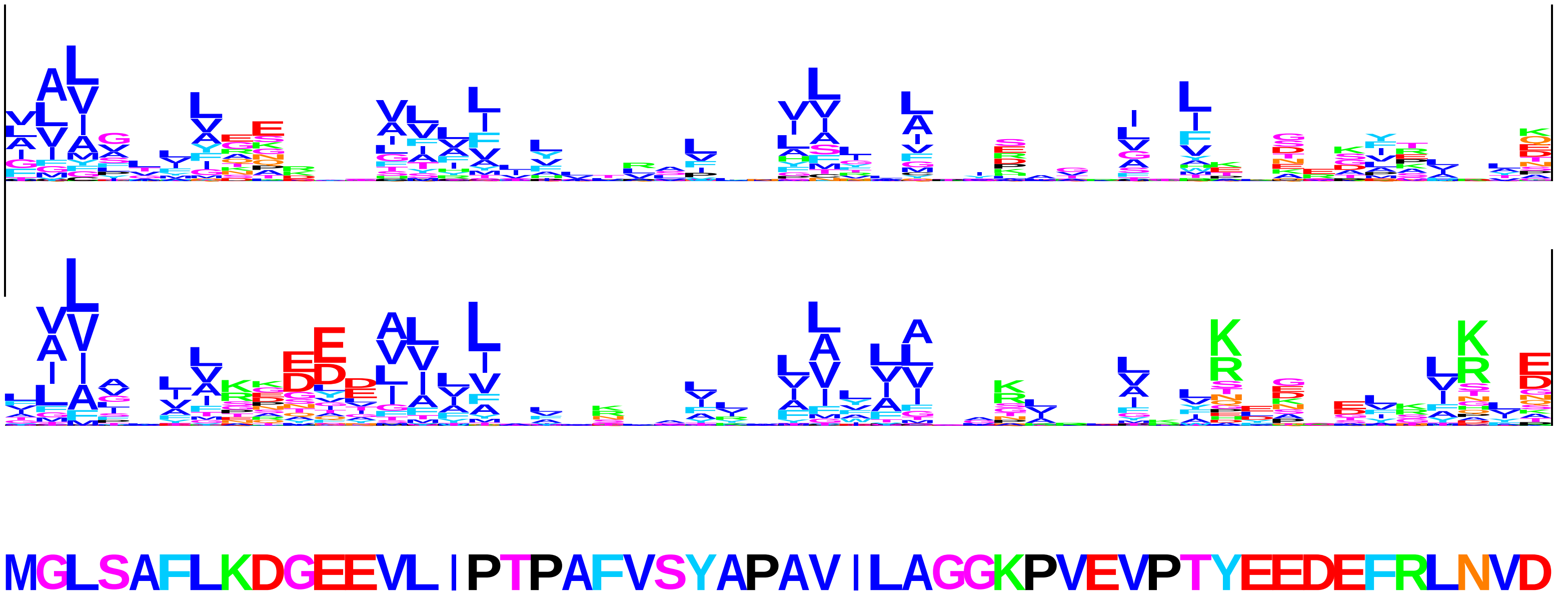
MGLSAFLKDGEEVLIPTPAFVSYAPAVILAGGKPVEVPTYEEDEFRLNVD

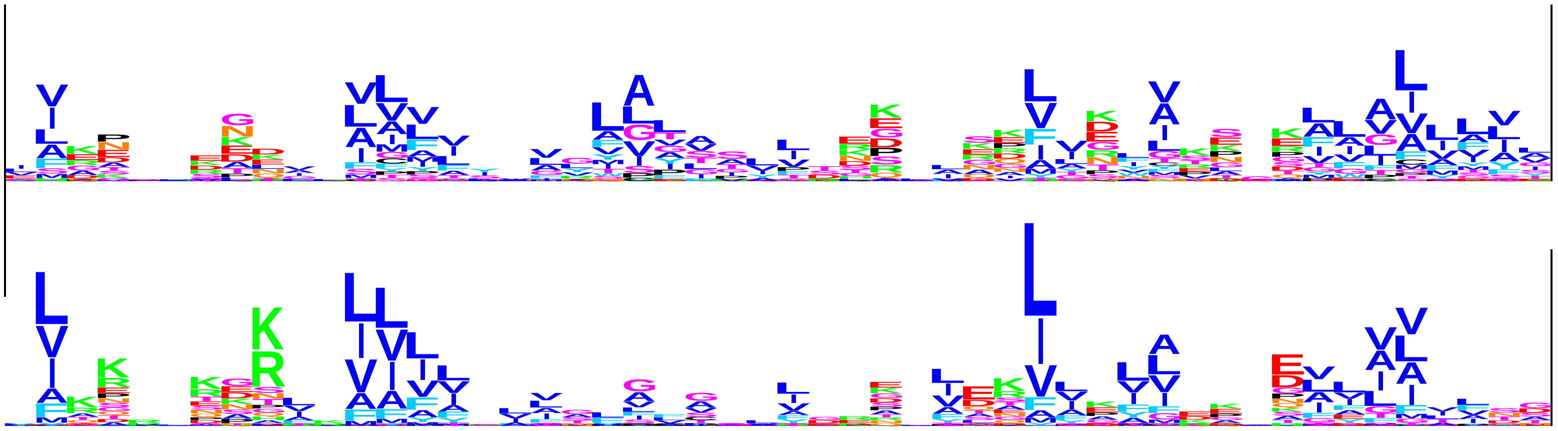

ELKKYVTDKTRALIINSPCNPTGAVLTKKDLEEIADFVVEHDLIVISDEV

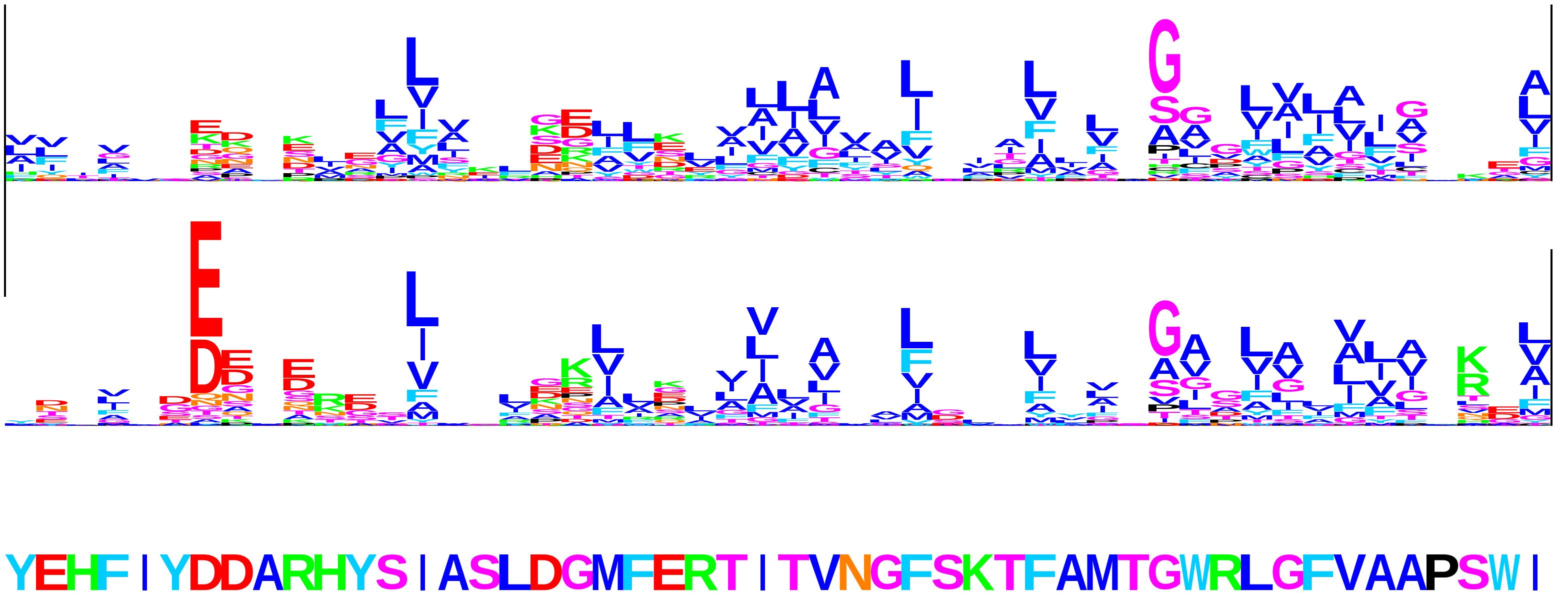
YEHFIYDDARHYSIASLDGMFERTITVNGFSKTFAMTGWRLGFVAAPSWI

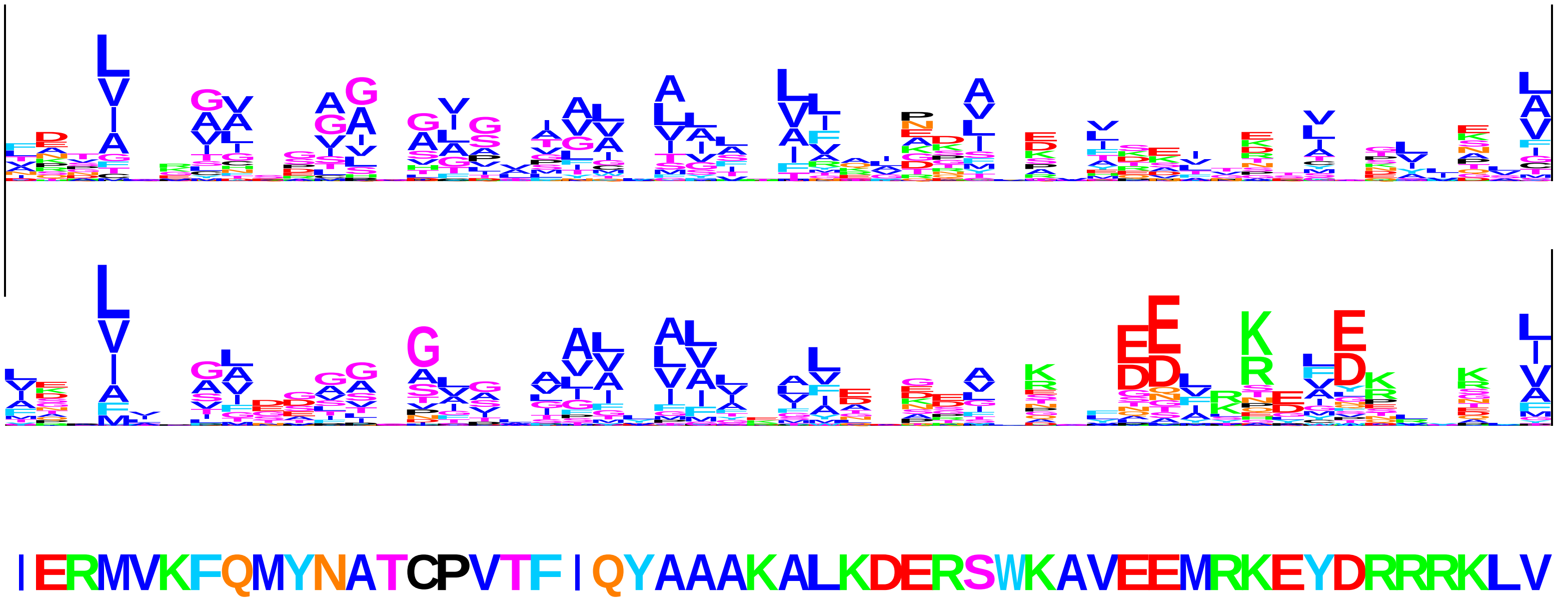
IERMVKFQMYNATCPVTFIQYAAAKALKDERSWKAVEEMRKEYDRRRKLV

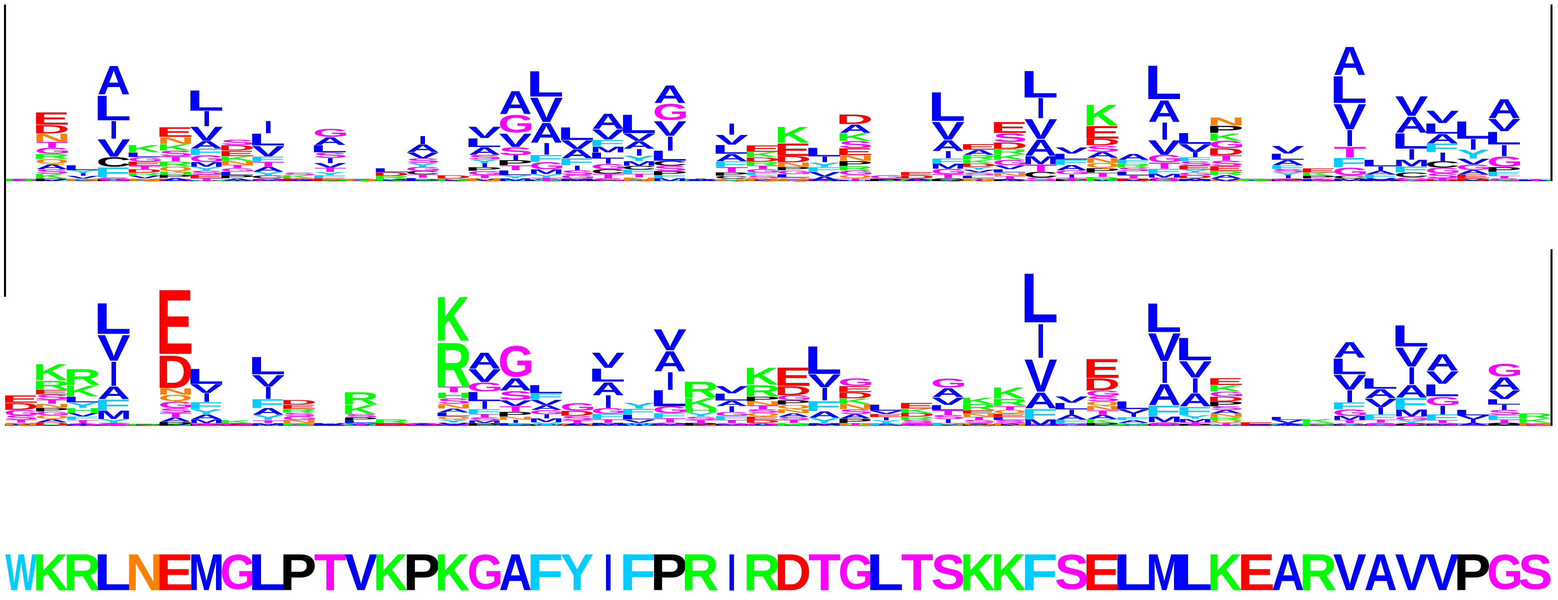
WKRLNEMGLPTVKPKGAFYIFPRIRDTGLTSKKFSELMLKEARVAVVPGS

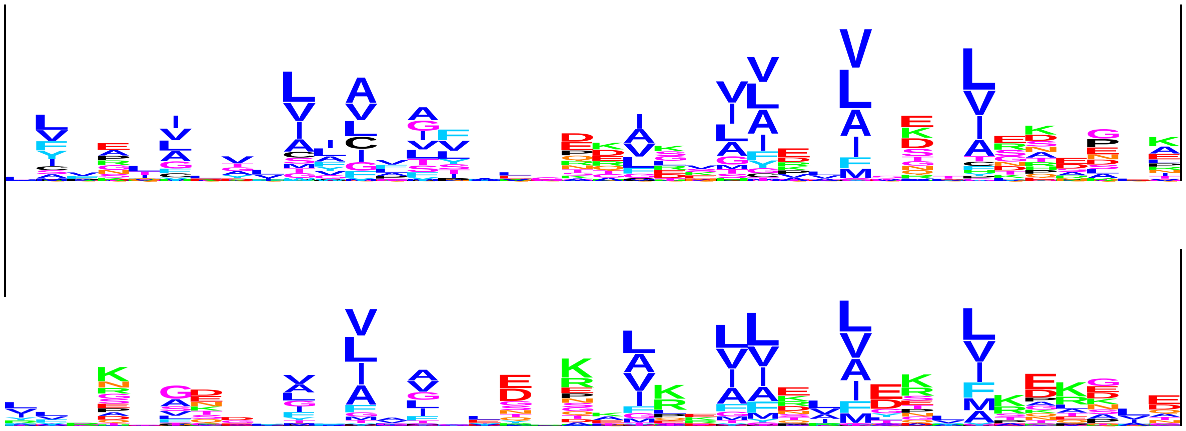

AFGKAGEGYVRISYATAYEKLEEAMDRMERVLKERKLV
