# Supplementary material for: A maximum likelihood framework for protein design
Source: BMC Bioinformatics. 2006 Jun 29;7:326. doi: 10.1186/1471-2105-7-326 (PMC1570151; doi:10.1186/1471-2105-7-326)
Supplement: Additional file 7 — Marginal and leave-one-out profiles of 10 proteins used in the design specificity experiment [file 1471-2105-7-326-S7.gz › 1LAY0.pdf]

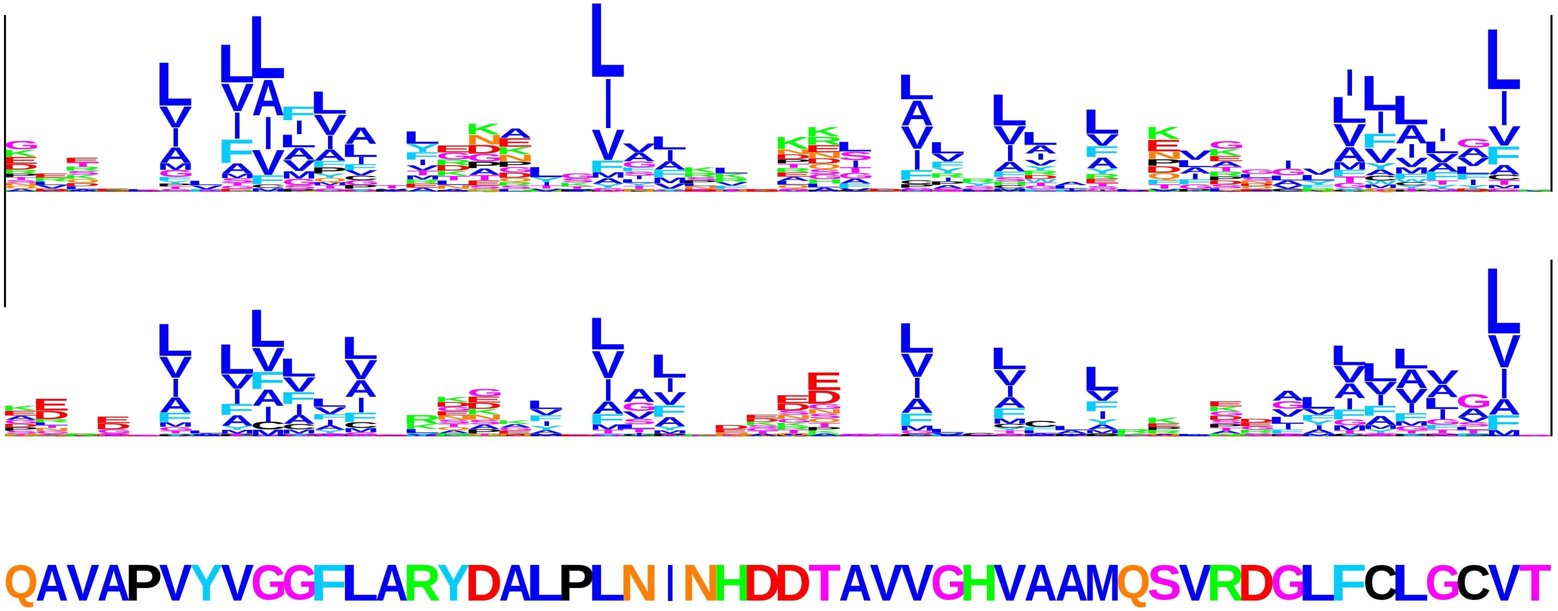
QAVAPVYVGGFLARYDALPLNINHDDTAVVGHVAAMQSVRDGLFCLGCVT

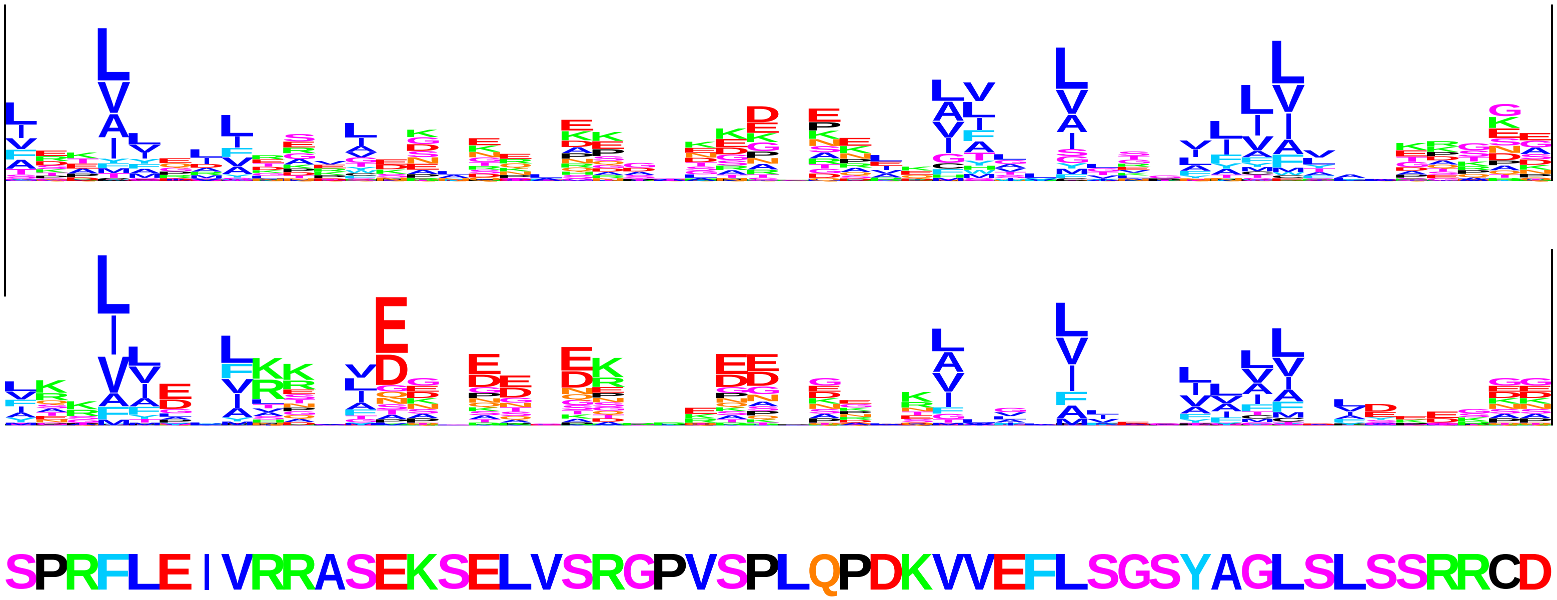
SPRFLEIVRRASEKSELVSRGPVSPLQPDKVVEFLSGSYAGLSLSSRRCD

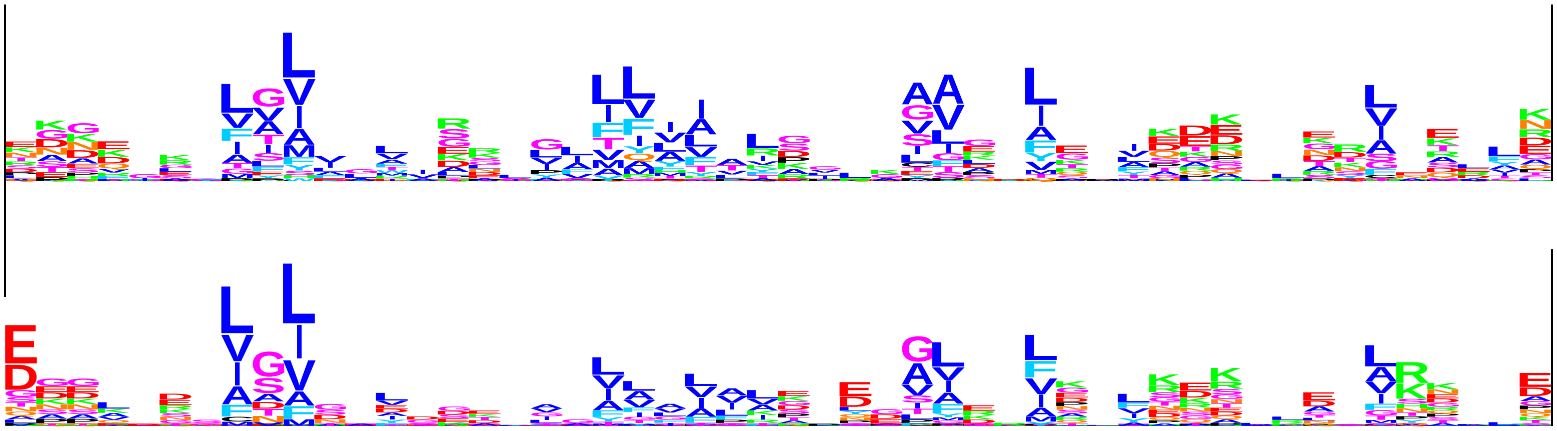

DVEPFKHVALCSVGRRRGTLAVYGRDPEWVTQRFPDLTAADRDGLRAQWQ

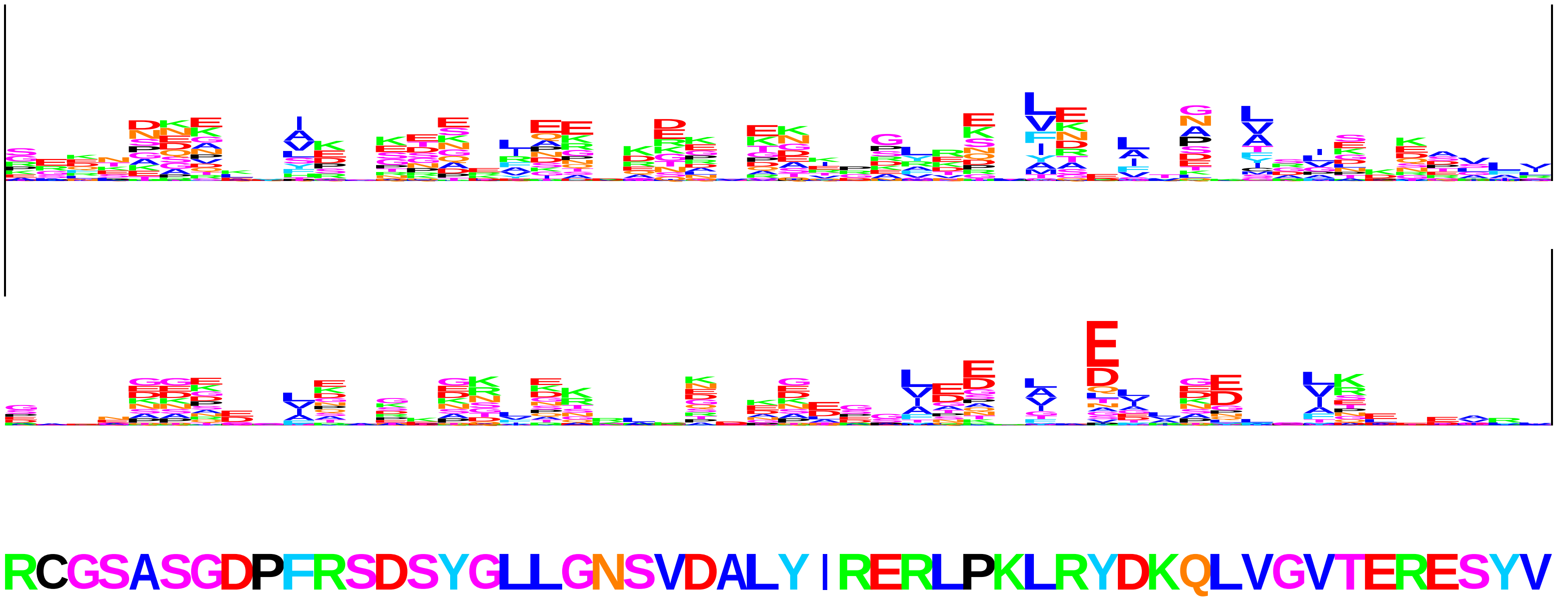
RCGSASGDPFRSDSYGLLGNSVDALYIRERLPKLRYDKQLVGVTERESYV

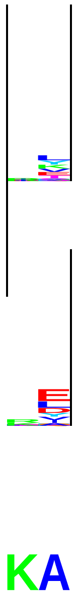
KA
