# Supplementary material for: A maximum likelihood framework for protein design
Source: BMC Bioinformatics. 2006 Jun 29;7:326. doi: 10.1186/1471-2105-7-326 (PMC1570151; doi:10.1186/1471-2105-7-326)
Supplement: Additional file 8 — Table 1: list of PDB identifiers of proteins used in the design specificity experiment, and scores obtained for each one of the proteins, using the combined (ε + α14ac + μ) potential [file 1471-2105-7-326-S8.pdf]

| PDB id | Average Z-score ratio | Ranking (median) | Target fold in top 1% | Target fold in top 10% | Average entropy/site | Average seq. Similarity | Average seq. Identity |
|---|---|---|---|---|---|---|---|
| 1EB6A | 0.8531 | 1 | 90% | 95% | 0.842 | 20.0 | 6.86 |
| 1TIYA | 0.7978 | 12.5 | 75% | 100% | 0.766 | 17.4 | 6.61 |
| 1B8XA | 0.7588 | 6.5 | 70% | 100% | 0.745 | 20.8 | 7.35 |
| 1VAVA | 0.7143 | 1 | 90% | 100% | 0.742 | 22.9 | 7.91 |
| 1LAY0 | 0.7503 | 2.5 | 90% | 100% | 0.805 | 22.2 | 8.69 |
| 2SCUA | 0.6885 | 1 | 90% | 100% | 0.838 | 22.5 | 8.37 |
| 1D2TA | 0.7174 | 2 | 90% | 100% | 0.932 | 21.0 | 7.66 |
| 1Q4MA | 0.5926 | 2.5 | 75% | 90% | 0.693 | 22.0 | 7.18 |
| 1B5L0 | 0.7181 | 2 | 80% | 100% | 0.766 | 25.4 | 8.85 |
| 1UMHA | 0.5743 | 50 | 50% | 75% | 0.712 | 21.0 | 7.58 |
| 1NNGA | 0.6736 | 2.5 | 85% | 95% | 0.755 | 22.7 | 7.16 |
| 1CE7A | 0.6346 | 11.5 | 75% | 90% | 0.769 | 21.4 | 7.95 |
| 2PTH0 | 0.6749 | 6 | 100% | 100% | 0.821 | 21.1 | 8.03 |
| 1NAL3 | 0.6030 | 2.5 | 90% | 100% | 0.845 | 24.4 | 8.49 |
| 1CTT0 | 0.6205 | 1 | 100% | 100% | 0.862 | 23.2 | 8.69 |
| 1GS5A | 0.5770 | 1 | 95% | 100% | 0.799 | 25.0 | 9.40 |
| 1DQYA | 0.6078 | 5.5 | 90% | 100% | 0.814 | 21.1 | 7.53 |
| 1C8OA | 0.5422 | 8 | 65% | 85% | 0.738 | 24.1 | 7.78 |
| 1VI9A | 0.5486 | 2.5 | 80% | 100% | 0.765 | 21.8 | 8.12 |
| 1CFZA | 0.5641 | 8.5 | 60% | 95% | 0.742 | 26.6 | 9.51 |
| 1UOX0 | 0.5280 | 5 | 65% | 90% | 0.700 | 21.4 | 7.34 |
| 1JJFA | 0.5664 | 6.5 | 80% | 100% | 0.782 | 21.6 | 8.25 |
| 1D2NA | 0.5389 | 5 | 85% | 100% | 0.767 | 26.2 | 8.72 |
| 1Q77A | 0.5027 | 6.5 | 65% | 85% | 0.691 | 25.5 | 8.94 |
| 3CLA0 | 0.5053 | 15 | 65% | 90% | 0.665 | 21.9 | 7.19 |
| 5NUL0 | 0.5286 | 2 | 75% | 100% | 0.787 | 26.6 | 8.22 |
| 1CV80 | 0.5249 | 14 | 60% | 100% | 0.737 | 22.2 | 7.04 |
| 1B9LA | 0.5373 | 48.5 | 50% | 75% | 0.716 | 22.3 | 7.02 |
| 1GHEA | 0.5285 | 71 | 45% | 80% | 0.650 | 21.4 | 7.98 |
| 1D4AA | 0.4495 | 78 | 35% | 75% | 0.725 | 21.8 | 8.32 |
| 1O70A | 0.4782 | 6 | 75% | 95% | 0.743 | 24.1 | 7.64 |
| 1MUN0 | 0.4379 | 40.5 | 50% | 75% | 0.762 | 21.6 | 7.47 |
| 1RXQA | 0.4393 | 37 | 55% | 80% | 0.725 | 24.2 | 8.25 |
| 1RIFA | 0.4916 | 6 | 95% | 95% | 0.725 | 21.1 | 6.68 |
| 1D2ZB | 0.4398 | 66.5 | 30% | 85% | 0.777 | 23.3 | 7.67 |
| 1HUW0 | 0.4336 | 98 | 35% | 80% | 0.734 | 23.4 | 7.14 |
| 3SDHA | 0.3600 | 113.5 | 40% | 75% | 0.709 | 25.6 | 8.69 |
| 1H31A | 0.4460 | 335 | 10% | 55% | 0.698 | 20.3 | 7.12 |
| 1EFDN | 0.3922 | 11 | 70% | 100% | 0.712 | 22.5 | 7.54 |
| 1JJVA | 0.3638 | 28.5 | 55% | 70% | 0.681 | 23.7 | 7.76 |
| 1RU8A | 0.3374 | 178.5 | 10% | 70% | 0.678 | 23.0 | 8.54 |
| 1B74A | 0.2983 | 89 | 50% | 53% | 0.801 | 26.0 | 9.35 |
| 1PA7A | 0.3455 | 147 | 21% | 58% | 0.704 | 22.3 | 7.28 |
| 1EF8A | 0.3580 | 95.5 | 30% | 85% | 0.778 | 22.6 | 7.87 |
| 1PQ4A | 0.3762 | 54 | 40% | 85% | 0.756 | 24.7 | 7.88 |
| 1ETB1 | 0.3255 | 274 | 30% | 55% | 0.702 | 21.0 | 8.69 |
| 1ETEA | 0.4506 | 405 | 40% | 50% | 0.717 | 23.1 | 7.31 |
| 3DFR0 | 0.3466 | 129 | 32% | 74% | 0.662 | 21.8 | 7.41 |

| | | | | | | | |
|---|---|---|---|---|---|---|---|
| 1UIZA | 0.3600 | 176 | 30% | 70% | 0.726 | 21.3 | 8.09 |
| 3TMKA | 0.3458 | 121.5 | 29% | 79% | 0.683 | 20.6 | 6.69 |
| 1AUVA | 0.3139 | 114.5 | 35% | 70% | 0.641 | 21.2 | 7.06 |
| 1GGGA | 0.2697 | 299.5 | 25% | 60% | 0.693 | 22.2 | 7.89 |
| 1F45B | 0.1965 | 736 | 16% | 32% | 0.687 | 21.6 | 6.64 |
| 2SAK0 | 0.1740 | 629.5 | 15% | 40% | 0.701 | 22.7 | 8.80 |
| 1R6FA | 0.1509 | 346 | 15% | 50% | 0.733 | 27.1 | 8.68 |
| 1T35A | 0.1739 | 1326 | 5% | 26% | 0.571 | 19.0 | 6.16 |
| 1KYQA | -0.3307 | 1056 | 8% | 28% | 0.660 | 21.3 | 6.97 |
| 1D9CA | 0.0005 | 1558 | 0% | 15% | 0.731 | 23.8 | 6.57 |
| 1QKRA | 0.0127 | 1878 | 5% | 15% | 0.727 | 23.7 | 7.76 |
| 1SFXA | -0.0508 | 2246 | 0% | 5% | 0.722 | 25.5 | 8.77 |
| **TOTAL** | **0.4526** | **32.75** | **53.6%** | **77.5%** | **0.738** | **22.7** | **7.82** |
